# Systematic assessment of diverse RNA modifications using nanopore direct RNA sequencing

**DOI:** 10.1093/nar/gkag411

**Published:** 2026-05-04

**Authors:** Xinqi Kang, Kelly Zhang, Alexandre Goyon, William Stephenson

**Affiliations:** Department of Synthetic Molecule Analytical Chemistry, Genentech, 1 DNA Way, South San Francisco, CA 94080, United States; Department of Synthetic Molecule Analytical Chemistry, Genentech, 1 DNA Way, South San Francisco, CA 94080, United States; Department of Synthetic Molecule Analytical Chemistry, Genentech, 1 DNA Way, South San Francisco, CA 94080, United States; Department of Proteomic and Genomic Technologies, Genentech, 1 DNA Way, South San Francisco, CA 94080, United States

## Abstract

While nanopore direct RNA sequencing has substantially advanced transcriptomics, its detection of RNA modifications remains primarily focused on abundant biological base modifications. However, therapeutic RNAs employ a diverse catalog of modifications, including base, sugar, and backbone modifications, to enhance stability and pharmacological properties. To address this gap, we systematically evaluated a set of therapeutically relevant modifications [phosphorothioate (PS)], sugar [2′-O-methylation (2′OMe), 2′-Fluoro (2′F), locked nucleic acid (LNA), 2′-O-(2′-methoxyethyl) (2′MOE)], and base [*N*^1^-methylpseudouridine (m1Ψ), 5-methylcytidine (m5C), 5-methoxyuridine (5moU), and 5-iodocytidine (5iodoC)] using direct RNA nanopore sequencing. Modifications were systematically analyzed using basecall errors, raw current signals, and modification-aware basecalling models. Ribose modifications, m1Ψ, and 5moU induced significant error rate increases and noticeable current alterations, whereas 2′OMe and 2′MOE affected dwell time adjacent to the pore. In contrast, PS linkages produced only slight current alterations without increasing basecalling errors. We further evaluated modification-aware basecallers for 2′OMe and m5C. While these tools can distinguish modification types, they are limited by poor quantification accuracy and high local error rates, especially for 2′OMe. This study establishes a critical performance baseline, clarifying the current capability and limitations of nanopore technology for the analysis of therapeutically relevant RNA modifications.

## Graphical Abstract

**Graphical Abstract:**
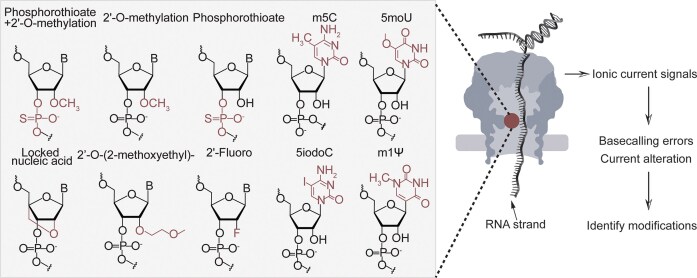

## Introduction

Over 170 RNA modifications have been identified to date, many of which play crucial roles in both natural biological processes and therapeutic applications [1–3]. Endogenous RNA modifications regulate structure, stability, and function, and their dysregulation is linked to a wide range of human diseases [2, 4]. In addition, dysregulation of the RNA modification machinery has been implicated in numerous disorders, including neurological disorders, autoimmune diseases, diabetes, and cancer [3, 4]. In the therapeutic context, chemical modifications have been applied to enhance the stability, delivery, and efficacy of RNA-based therapeutics while reducing their immunogenicity [5, 6].

Nanopore sequencing technology offers single-molecule resolution and, crucially, direct sequencing of native RNA molecules without the need for reverse transcription or amplification, which allows for the potential simultaneous detection of multiple modification types on a single transcript [7–10]. Furthermore, direct RNA sequencing can produce multi-kilobase read lengths [11], which are advantageous for resolving transcript isoforms due to alternative splicing and alternative transcription. This capability is also well-suited in the therapeutic context for characterizing large molecules like messenger RNAs (mRNAs) by assessing full-length sequence integrity, estimating poly(A) tail length, and evaluating purity [12, 13]. While therapeutic RNA characterization via direct RNA nanopore sequencing has traditionally focused on mRNAs, recent software advancements now enable the analysis of shorter transcripts in the 70–90 nt range [14, 15].

Most direct RNA nanopore sequencing studies have focused on transcriptome analysis [10, 16–18], primarily prioritizing the detection of abundant base modifications, such as *N*^6^-methyladenosine (m6A) [16] for mRNA stability [19] and pseudouridine (Ψ) [20–23] for RNA structure [24], while investigations into sugar modifications have been limited, with only a small number of studies addressing 2′-O-methylation (2′OMe) [25–29]. Despite dedicated efforts to detect these modifications, most available data rely on legacy Oxford Nanopore chemistries and basecallers, which yielded an accuracy of ~90% or lower [8, 30]. Although Oxford Nanopore has recently introduced updated direct RNA sequencing chemistry and basecallers claiming significant improvements in accuracy, independent validation remains limited, and a key concern is that existing detection algorithms may not be fully compatible with these updates [31]. As of March 2026, nanopore direct RNA sequencing offers modification-aware basecallers for only a handful of modifications, including m6A, Inosine (I), 5-methylcytidine (m5C), Ψ, and 2′OMe [32]. Even with this notable step forward, a substantial number of known RNA modifications still lack dedicated basecallers. A fundamental question persists regarding whether all distinct types of RNA modifications, spanning base, sugar, and particularly the unstudied phosphate backbone, elicit sufficiently distinct current signals to be resolved during nanopore translocation [33].

The knowledge gap is even more pronounced in the direct analysis of therapeutic RNAs, where a diverse array of chemical modifications are utilized. Phosphorothioate (PS) linkages are commonly incorporated into various oligonucleotide classes, including FDA-approved drugs like the antisense oligonucleotide (ASO) Fomivirsen and the small interfering RNA (siRNA) Givosiran. Beyond phosphate backbone modifications, most approved ASOs and siRNAs utilized sugar modifications. For instance, 2′-O-(2′-methoxyethyl) (2′MOE) sugars in the ASO Mipomersen and a more complex mix of 2′OMe and 2′-Fluoro (2′F) along with an N-acetylgalactosamine moiety in the siRNA Givosiran [34, 35]. Furthermore, a rapidly increasing diversity of modifications is being evaluated in the clinic and at the research stage, creating an urgent need for more advanced sequencing tools. The success of *N*^1^-methylpseudouridine (m1Ψ) in mRNA vaccines, recognized by the 2023 Nobel Prize [36], has accelerated this trend. Despite the critical importance of verifying these modifications, there is a striking lack of published studies applying nanopore sequencing to characterize therapeutic RNAs and the chemical modifications common to them. The VAX-seq study, for example, successfully applied nanopore sequencing to assess key quality attributes of mRNA vaccines and showed that m1Ψ presence correlates with an increased basecalling error rate [37]. While this provides a valuable method for detection, a more quantitative approach would be beneficial for robust characterization.

This study aims to address these critical gaps by systematically probing the capabilities of the latest nanopore direct RNA sequencing chemistry (RNA004) to detect a diverse range of therapeutically and biologically relevant base, sugar, and phosphate backbone modifications. For those modifications that lacked dedicated basecallers, our approach involved the application of total error rate and current analysis. Additionally, for modifications where specific basecallers were available, we leveraged these validated tools to assess detection performance. This work provides a performance benchmark for future applications in the quality control of therapeutic RNA, highlighting capabilities and limitations of direct RNA nanopore sequencing for detecting therapeutically relevant modifications.

## Materials and methods

### PolyA tailing

Site-specifically modified 120-nt synthetic RNAs—including canonical, Chimera-(PS/2′OMe), Chimera-(LNA/2′MOE/2′F), and 2′OMePS variants—were produced via solid-phase synthesis and HPLC-purified by Integrated DNA Technologies. For polyadenylation, 1 µg of each RNA oligonucleotide was prepared in 15 µl of nuclease-free water. The poly(A) tailing reaction was assembled by combining the RNA with 2 µl of 10× *Escherichia coli* poly(A) polymerase reaction buffer (NEB, B0276SVIAL), 2 µl of 10 mM ATP (NEB, B0756AVIAL), 1 µl of *E. coli* poly(A) polymerase (NEB, M0276), and 0.5 µl of murine RNase inhibitor (NEB, M0314) to enhance RNA stability. The reaction volume was adjusted to a final total of 20 µl with nuclease-free water. Samples were incubated at 37°C for 30 min. Following the tailing reaction, RNA was purified using the Zymo RNA Clean & Concentrator-5 kit (Zymo Research, R1014). Polyadenylated RNA was first brought to 50 µl with nuclease-free water, then mixed with 100 µl RNA Binding Buffer and 150 µl of 100% ethanol. The mixture was loaded onto Zymo-Spin™ IC columns and washed sequentially with RNA Prep Buffer and RNA Wash Buffer according to the manufacturer’s instructions. RNA was eluted in 15 µl of nuclease-free water by centrifugation at 10 000–16 000 × *g* for 30 s. Final RNA concentrations were measured using a Nanodrop spectrophotometer, and poly(A) tail lengths were estimated using the Agilent TapeStation RNA ScreenTape (5067–5576 RNA ScreenTape, 5067–5578 RNA ScreenTape ladder, 5067–5577 RNA ScreenTape sample buffer), following the manufacturer’s protocol.

### Direct RNA sequencing

mRNAs used in this study, including canonical mCherry and eGFP mRNAs, modified mCherry and eGFP mRNAs with m1Ψ or 5moU or 5iodoC or m5C, were produced via *in vitro* transcription (IVT) and oligo(dT)-purified by GenScript. Before library preparation, site-specifically modified 120-nt synthetic RNAs underwent polyadenylation and subsequent library preparation, with each sequence then loaded into a single flow cell. For mRNA sequencing, mCherry and eGFP with the same nucleobase modification were mixed in a 1:1 ratio prior to library preparation (e.g. canonical mCherry and eGFP were mixed in a 1:1 ratio, or m1Ψ mCherry and eGFP were mixed in a 1:1 ratio). Direct RNA libraries were prepared using the ONT Direct RNA Sequencing Kit (SQK-RNA004) following the manufacturer’s standard protocol. The workflow comprised RT adapter ligation, reverse transcription and purification, sequencing adapter ligation and purification, and flow cell priming and loading. All surfaces and equipment were decontaminated with RNaseZAP before use. For RT adapter ligation, 300 ng of poly(A)-tailed RNA was diluted to 8 µl with nuclease-free water and combined with 3 µl NEBNext^®^ Quick Ligation Reaction Buffer (NEB, B6058), 1 µl murine RNase inhibitor (NEB, M0314), 1 µl RT adapter (ONT, SQK-RNA004), and 1.5 µl T4 DNA ligase (2 million U/ml; NEB, M0202M) to a total volume of 15 µl. The mixture was gently mixed, briefly centrifuged, and incubated at room temperature for 10 min. Reverse transcription was performed by adding a master mix of nuclease-free water, dNTPs (NEB, N0447), and Induro^®^ RT Reaction Buffer (NEB, B0681AVIAL), followed by Induro^®^ Reverse Transcriptase (NEB, M0681), and incubated at 60°C for 30 min, then 70°C for 10 min. The reverse transcription product was purified with Agencourt RNAClean XP beads (Beckman Coulter™, A63987) using 70% ethanol washes and eluted in 23 µl nuclease-free water. Sequencing adapter ligation was performed by mixing 23 µl cDNA, 8 µl NEBNext^®^ Quick Ligation Reaction Buffer, 6 µl RNA ligation adapter (ONT, SQK-RNA004), and 3 µl T4 DNA ligase, incubated for 10 min at room temperature. The ligation product was purified using Agencourt RNAClean XP beads with ONT Wash Buffer, followed by elution in RNA Elution Buffer (ONT, SQK-RNA004). Final libraries were quantified with Qubit fluorometry, targeting >30 ng recovery, and loaded onto flow cells following ONT priming and loading procedures, ensuring careful removal of air bubbles to preserve pore integrity and sequencing performance. All sequencing runs were performed on MinION flow cells, with the exception of canonical mRNAs (canonical mCherry and eGFP in 1:1 ratio mix), which were run on a PromethION flow cell. Sequencing runs were controlled via MinKNOW software (v 25.03.7) using the direct RNA protocol.

#### Data analysis

##### Basecalling and alignment

To analyze nanopore direct RNA sequencing data, Dorado (v1.0.0) was used for basecalling, specifically employing the rna004_130bps_hac@v3.0.1 model unless otherwise stated. Move table information was also emitted during basecalling using the --emit-moves flag. Following basecalling, Minimap2 (v2.24) facilitated read alignment. RNA-specific alignment was performed using the command minimap2 -ax splice -uf -k14 --MD, ensuring the preservation of modification-related tags (MM, ML, mv, ts, pi, sp, ns). For mRNAs, alignment was restricted to the mCherry and eGFP coding sequences only, due to proprietary 3′ and 5′ UTR information. The resulting aligned BAM files were then sorted and indexed using Samtools (v1.21). Finally, a filtering step with Samtools removed secondary and unmapped reads, retaining only uniquely mapped reads with a minimum mapping quality of Q20. These filtered BAM files were subsequently sorted and indexed for downstream analysis.

##### Total variation analysis

Total variation analysis was conducted using pysam (v0.22.1) to perform a pileup analysis on the BAM files generated during basecalling and alignment. For each genomic position, the script quantified the frequency of all observed bases (A, T, G, C), as well as insertions and deletions. This raw data were then exported to a CSV file. Subsequently, the script processed this information to determine the total variation percentage, defined as the percentage of total non-reference events (Mismatched Base Calls + Deletion Calls + Insertion Calls) at a given position. Plots visualizing sequencing depth and coverage across the reference were also generated. The total variation percentage is calculated as:


\begin{eqnarray*}&&\textit{Total}\ \textit{variation}\ \textit{percentage} \\&=& \ \frac{{\textit{Mismatched}\ \textit{Base}\ \textit{Calls}\ + \textit{Deletion}\ \textit{Calls}\ + \ \textit{Insertion}\ \textit{Calls}}}{{A\ \textit{calls}\ + T\ \textit{calls}\ + G\ \textit{calls}\ + C\ \textit{calls}\ + \textit{deletion}\ \textit{calls}\ + \textit{insertion}\ \textit{calls}}}.\end{eqnarray*}

##### Current analysis

Our current analysis initiates by extracting per-read, per-base signal matrices—comprising dwell time, current mean, and current standard deviation—followed by energy distance calculation, a multivariate nonparametric analysis. Signal matrix extraction was performed using either Remora (v3.3.0) [38] or Uncalled4 (v4.1.1) [39]. For both approaches, mRNA-aligned reads were filtered to retain only those with full coverage above 90%, conserving memory.

When using Remora, aligned reads and raw signal (POD5) data were aligned and refined using the SigMapRefiner module. This refinement primarily aims to align observed signal levels with expected base-specific signal levels. Specifically, do_rough_rescale = True and scale_iters = 0 were set. This configuration performs an initial, approximate signal scaling by comparing observed signal quantiles (from the basecaller’s move table) to expected k-mer signal quantiles, bringing the overall signal closer to the expected range without iterative re-scaling. The do_fix_gauge = True option was also enabled, normalizing expected signal values to a robust mean of ~0 and a standard deviation of 1 using median and median absolute deviation. Following this refinement, signal features (dwell time, current mean, and current standard deviation) were extracted for both modified and unmodified (control) RNA samples across k-mer windows along the reference. The current mean and standard deviation here refer to the trimmed mean and standard deviation of signal points assigned to each base, where a fixed number of signal points from the start and end of the base are trimmed using Remora’s default settings.

For signal matrix extraction with Uncalled4, the same BAM and POD5 files were utilized as with Remora. Uncalled4’s alignment function was employed, with corresponding flow cell and kit information inputted to extract the dwell time, current mean, and current standard deviation per read per base.

Finally, the signal matrices from both Remora and Uncalled4 were subjected to energy distance calculation. This calculation incorporated three signal metrics—dwell time, current mean, and current standard deviation—for the current base, along with one upstream and one downstream base, resulting in a 9-dimensional feature space. Prior to computation, dwell time data underwent log-transformation, and all signal metrics were robustly scaled. The following is the equation used for energy distance:


\begin{eqnarray*}
{{E}_{n,m}}\ \left( {X,Y} \right) &=& \ \frac{2}{{nm}}\ \mathop \sum \limits_{i = 1}^n \mathop \sum \limits_{j = 1}^m \left| {\left| {{{x}_{i\ }} - {{y}_i}} \right|} \right|\ \\&&- \ \frac{1}{{{{n}^{2\ }}}}\mathop \sum \limits_{i = 1}^n \mathop \sum \limits_{j = 1}^n \left| {\left| {{{x}_{i\ }} - {{y}_j}} \right|} \right| - \frac{1}{{{{m}^{2\ }}}}\mathop \sum \limits_{i = 1}^m \mathop \sum \limits_{j = 1}^m \left| {\left| {{{y}_{i\ }} - {{y}_j}} \right|} \right|
\end{eqnarray*}

$X$ and $Y$: sets of 9-dimensional feature vectors from the modified and unmodified samples, respectively.

$n$ and $m$: the number of reads in the modified and unmodified samples.

${{x}_{i\ }}\ $ and ${{y}_j}$ : the individual 9-dimensional feature vectors.

$| {| {} |} |$ : the Euclidean distance between two vectors.

##### Modification-aware basecaller

rna004_130bps_sup@v5.2.0_inosine_m6A_2OmeA@v1 model was used for Chimera-(PS/2′OMe) basecalling. rna004_130bps_sup@v5.2.0_inosine_m6A_2OmeA@v1, rna004_130bps_sup@v5.2.0_m5C_2OmeC@v1, rna004_130bps_sup@v5.2.0_pseU_2OmeU@v1 were used for canonical control and Cluster-2′OMePS basecalling. For m5C mCherry and eGFP, rna004_130bps_sup@v5.1.0_m5C@v1 and rna004_130bps_sup@v5.2.0_m5C_2OmeC@v1 were used. All data followed the same Minimap2 alignment procedure; reads underwent filtering to remove secondary and unmapped alignments, retaining only uniquely mapped reads with a minimum mapping quality of Q20, the same as described earlier. Subsequently, Modkit was utilized to pile up the modified bases specified by the basecalling model. The resulting data were then visualized as bar plots depicting the percentage of modified bases.

### Results

I. RNA modification types and analysis strategies

**Figure 1.**
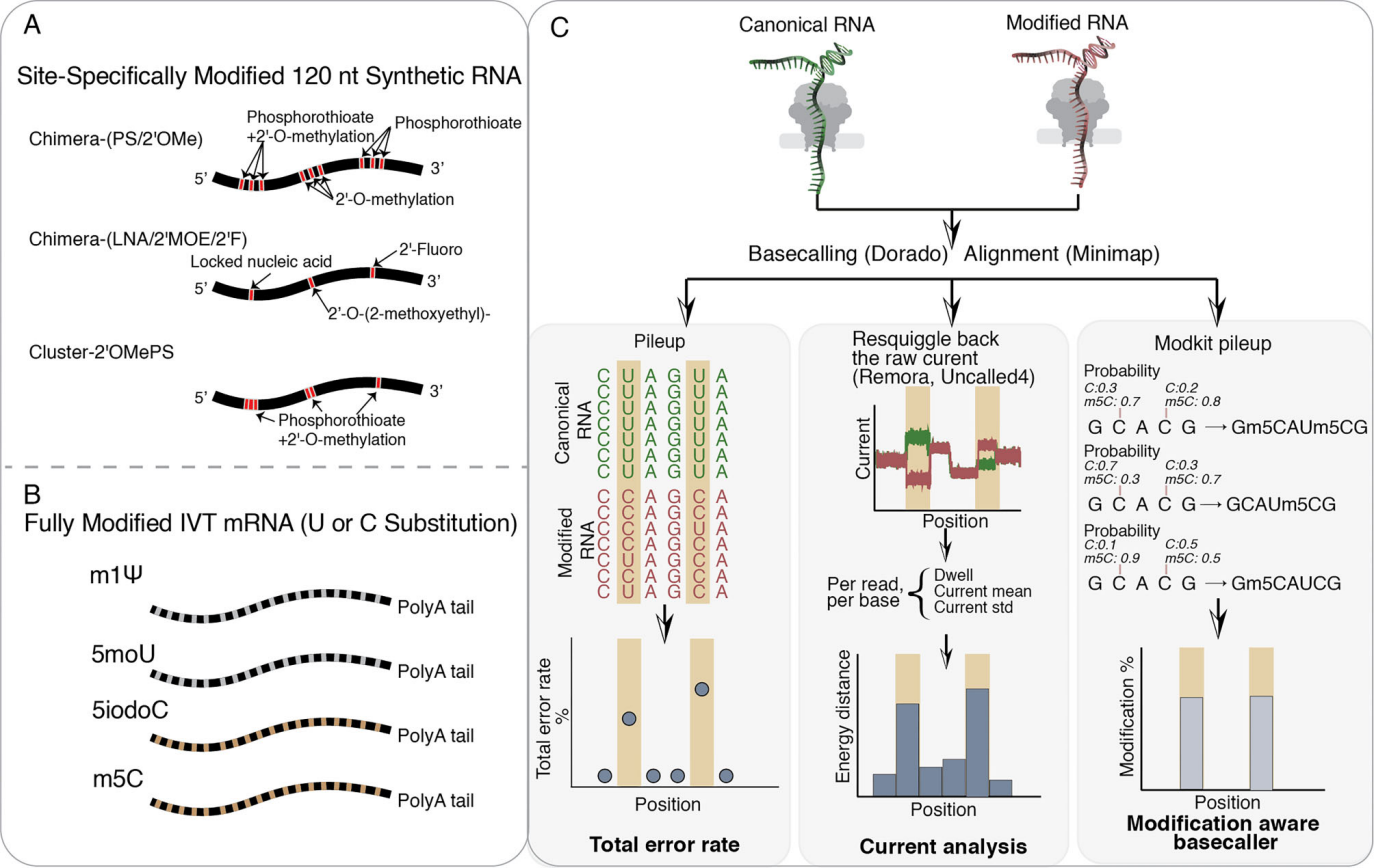
Overview of modified RNA constructs and detection strategy. (**A**) Site-specific phosphate backbone and ribose sugar modifications tested using three synthetic 120-nt RNAs—Chimera-(PS/2′OMe), Chimera-(LNA/2′MOE/2′F), and Cluster-2′OMePS—with identical base sequences but distinct phosphate or sugar chemistries. Modification sites are at positions 33–37, 70–74, and 107–111. (**B**) Fully substituted base modifications tested using mCherry and eGFP mRNAs. m1Ψ and 5moU fully replace U, while 5iodoC and m5C fully replace C. (**C**) Workflow for detecting RNA modifications with and without modification-aware basecallers. Modified RNAs and their canonical controls were sequenced. For modifications lacking modification-aware basecallers, both total error rate and current-level analyses were applied.

Synthetic siRNAs, ASOs, and guide RNAs (gRNAs) rely extensively on phosphorothioate (PS) backbones and sugar modifications (such as 2′OMe, 2′F, LNA, and 2′MOE). In the context of siRNAs and ASOs, they improve pharmacokinetic and pharmacodynamic properties by enhancing resistance against nucleases [6]. For CRISPR–Cas systems, these gRNA modifications further refine editing precision by increasing on-target activity while minimizing off-target effects [5]. In contrast, IVT-produced mRNAs incorporate base modifications like N1-methylpseudouridine (m1Ψ) to modulate immunogenicity [40]. To systematically evaluate the detection capabilities of nanopore direct RNA sequencing, we selected a panel of therapeutically relevant modifications. Model oligonucleotides containing specific phosphate and sugar modifications were generated via solid-phase synthesis, while IVT-produced mRNAs incorporating various modified nucleosides were obtained to assess base-level modifications. Phosphate backbone and sugar modifications were tested using three site-specifically modified 120-nt synthetic RNAs: Chimera-(PS/2′OMe), Chimera-(LNA/2′MOE/2′F), and Cluster-2′OMePS (Fig. 1A). All three constructs share the same base sequence but differ in sugar or backbone chemistry, with modifications located at positions 33–37, 70–74, and 107–111. This design allows us to benchmark sensing limits: isolated modifications assess sensitivity, while clustered patterns challenge the pore’s spatial resolution to mimic high-density therapeutic gRNA termini. For base modifications, we used mCherry and eGFP mRNAs in which m1Ψ and 5moU fully replace U, and 5iodoC and m5C fully replace C (Fig. 1B). We acknowledge that these synthetic and IVT constructs represent a simplified system compared to complex therapeutic RNAs and native transcriptome samples. Nevertheless, this simplification is crucial as it establishes a necessary baseline, offering an advantage over much of the published data derived from transcriptome samples with unknown modification stoichiometry. Detailed sequence information is provided in Supplementary Text S1.

Since most therapeutic RNA modifications currently lack trained modification-aware basecallers, we evaluated their detectability using two complementary approaches: total error rate and raw current analysis (Fig. 1C). We utilized the total error rate as a primary screening metric. Dorado-basecalled [32] reads were aligned to the reference with minimap2 [41], and we calculated the percentage of all non-reference events, including base mismatches, insertions, and deletions (“Materials and methods” section). Although high error rates often signal the presence of a modification, this metric relies heavily on the basecaller’s interpretation. Consequently, this reliance on basecaller output can mask subtle modifications that perturb the underlying ionic current but fall below the threshold for a detectable basecalling error.

To characterize the physical interaction between RNA and nanopore system independent of basecalling, we applied energy distance analysis to our synthetic constructs. Unlike transcriptomic studies limited by variable stoichiometry, our controlled design directly links signal perturbations to specific chemistries. We extracted three per-read, per-position features: dwell time (reflecting nucleotide-nanopore interaction and motor kinetics), current mean (pore blockage), and current standard deviation. Since individual features cannot fully explain signal perturbations, we computed the energy distance across sliding 3-nt windows. This non-parametric, multivariate metric integrates non-Gaussian distributions from all three features, capturing the aggregate physical impact of the modification on the nanopore sensing environment [42, 43]. While the emergence of new modification-aware basecalling models offers exciting capabilities, their reliability for quantification and basecalling accuracy in therapeutic applications lacks sufficient validation. To address this, we applied the appropriate models and Modkit [44] to pure synthetic standards, computing the percentage of modified calls per position. This approach complements recent biological benchmarks [45] by rigorously testing the platform’s ability to verify modification of stoichiometry for therapeutic quality control.

II. Differential impact of 2′OMe and PS modifications  

**Figure 2.**
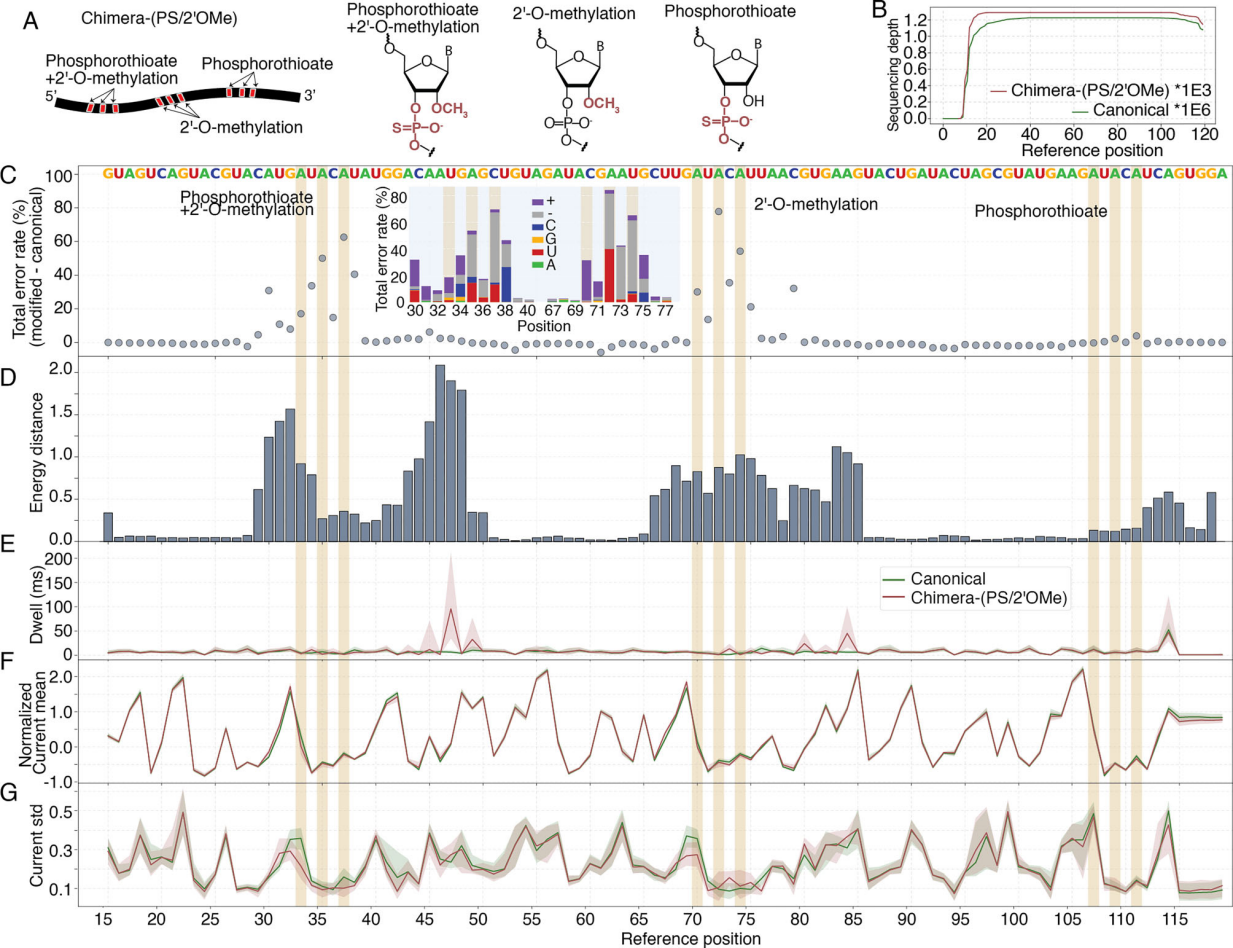
Error and current signatures of RNA modifications in Chimera-(PS/2′OMe). (**A**) Schematic of Chimera-(PS/2′OMe) showing clustered PS, 2′OMe, and 2′OMePS modifications. Chemical structures are shown alongside. (**B**) Sequencing depth of canonical and modified RNA. Canonical RNA has ~1.2 × 10⁶ reads, while Chimera-(PS/2′OMe) has ~1.2 × 10³. Both show decreased coverage at the 5′ end due to sequencing directionality and motor protein limitations. (**C**) Total error rate difference between Chimera-(PS/2′OMe) and canonical RNA. Elevated total error rates are observed at and near 2′OMe and 2′OMePS sites. PS modifications show minimal impact. The inset shows a stacked box plot of the total error rate, with each stack representing a different type of error percentage. (**D**) Energy distance per position between canonical and Chimera-(PS/2′OMe). Strong signals are seen at 2′OMe and 2′OMePS sites, with broader around 10 nt impact zones. Breakdown of current-level metrics: (**E**) Dwell time, (**F**) Normalized current mean, and (**G**) Current standard deviation. In all dwell time, current mean, and current standard deviation plots, the solid line represents the median across all reads, with the shaded area indicating the 25th to 75th percentile range. The highlighted regions indicate the modification sites, and the letters above the total error rate plots represent the reference sequence.

In order to explore the capability of nanopore sequencing to detect backbone modifications, we generated a construct [Chimera-(PS/2′OMe)] containing clusters of PS, 2′OMe, and 2′OMePS modifications, each sharing the same base—adenosine (Fig. 2A). Each cluster includes three modified positions: 33, 35, and 37 for 2′OMePS; 70, 72, and 74 for 2′OMe; and 107, 109, and 111 for PS. To ensure compatibility of the construct with the direct RNA nanopore sequencing library preparation kit, we added synthetic poly(A) tails using *E. coli* poly(A) polymerase (Supplementary Fig. S1). The canonical construct exhibited higher sequencing depth between reference positions 20–120 compared to the modification-containing Chimera-(PS/2′OMe) (~1.2 × 10⁶ versus ~1.2 × 10³ number of reads, respectively) (Fig. 2B). For both the canonical and Chimera-(PS/2′OMe), few reads cover the 5′ end (~500 reads for Chimera-(PS/2′OMe) at position 5 and 6, then drop down to <5 reads at position 1 and 2), which is expected, since nanopore direct RNA sequencing begins at the 3′ end and often lacks sufficient coverage at the final 10 nt of the 5′ end where the motor protein fails to maintain grip. Therapeutic modifications such as PS, LNA, and 2′F are unreported in direct RNA nanopore sequencing literature; their impact on pore translocation and motor protein function remains unknown. The 1000-fold difference in depth between canonical and Chimera-(PS/2′OMe) may be explained by unfavorable interactions between the 2′OMe and PS with the motor protein or pore. These interactions could plausibly hinder the RNA’s translocation through the pore and increase error rates in the resulting reads, preventing proper alignment to the reference. Although such read reduction poses a risk of low statistical power for future studies, this specific work retained sufficient coverage. When the same Chimera-(PS/2′OMe) reads are basecalled with a modification-aware basecaller and aligned using the same minimap2 workflow, sequencing depth increases up to 42 718 (Supplementary Fig. S9). Nevertheless, all three modified regions in Chimera-(PS/2′OMe) still show >1000 aligned reads, providing sufficient sequencing coverage for analysis.

We next performed total error rate analysis for both the canonical and Chimera-(PS/2′OMe) constructs. The canonical control exhibited an overall error rate <5% in the majority of positions (Supplementary Fig. S2). The 2′OMePS and 2′OMe modifications induced elevated total error rates at both the modified sites and proximal bases (Fig. 2C). For most of the 2′OMePS and 2′OMe positions, the error rate exceeded 20%. In contrast, unmodified positions maintained an error rate below 5%. For the sequence contexts tested here, these modifications appear to preferentially cause deletions (Fig. 2C inset).

This disruption in basecalling accuracy is also captured by the energy distance metric, which measures the dissimilarity in the raw electrical signal compared to the canonical sequence. Interestingly, the energy distance often reveals a broader footprint of disruption than error rates alone (Fig. 2D), extending significantly downstream from the actual modification site. This offset is likely a consequence of the 3′-to-5′ sequencing direction, potentially reflecting the physical distance between the motor protein and the pore’s constriction where the signal is read. As the pore analyzes a specific nucleotide, the motor protein has already encountered structural changes several nucleotides upstream, and we hypothesize that this earlier interaction may perturb the signal, which is consistent with other reports in the literature [21, 24]. For example, when sequencing position 84, the 2′OMe at position 74 resides near the motor protein, causing current discrepancies, more specifically, a dwell time discrepancy. Notably, several regions (e.g. positions 29–48 and 66–85) showed high energy distance without a corresponding increase in error rates. This suggests that while these modifications produce a distinct signal disturbance, the Dorado basecaller can often overcome this perturbation to correctly identify the base, separating signal disruption from sequencing error.

Energy distance was calculated using per-read, per-base current metrics: dwell time (Fig. 2E), normalized current mean (Fig. 2F), and current standard deviation (Fig. 2G). There is an increase in dwell time ~10 nt upstream of the 2′OMePS and 2′OMe sites (position 47 and position 84). The bulky methyl group is known to induce a rigid C3′-endo ribose sugar conformation [46, 47], and we speculate that this altered structure or the resulting steric interactions with the motor protein may be the cause of the delay in translocation. Differences in the current standard deviation were observed between the modified and canonical RNA at both 2′OMePS (positions 30–48) and 2′OMe (positions 67–77) sites (Fig. 2G). This difference may suggest an interaction between the methyl groups and the nanopore wall.

In contrast to the substantial increase in sequencing errors induced by the 2′OMe ribose modifications, the PS-modified sites exhibited high basecalling fidelity, with total error rates remaining near the baseline of the canonical control (Fig. 2C). Analysis using the energy distance metric revealed a modest peak corresponding to the PS modified region (Fig. 2D). This elevation in energy distance, which was not apparent in the error rate analysis, indicates that the modification does produce a subtle perturbation of the ionic current, albeit one insufficient to cause a misclassification of the base by the basecaller. Dwell time remained largely unaffected, suggesting that the PS modification likely does not impede the translocation process. As the PS substitution induces only moderate shifts in charge and size, its physical interactions with the motor protein and nanopore wall appear comparable to canonical RNA. It is important to note that PS was the only phosphate backbone modification evaluated in this study due to commercial availability. Consequently, generalization of this minimal signal perturbation to all possible backbone chemistries is limited, as more structurally diverse modifications, such as phosphorodithioate, may potentially elicit a stronger nanopore signal.

III. Characterizing LNA, 2′MOE, and 2′F modifications

**Figure 3.**
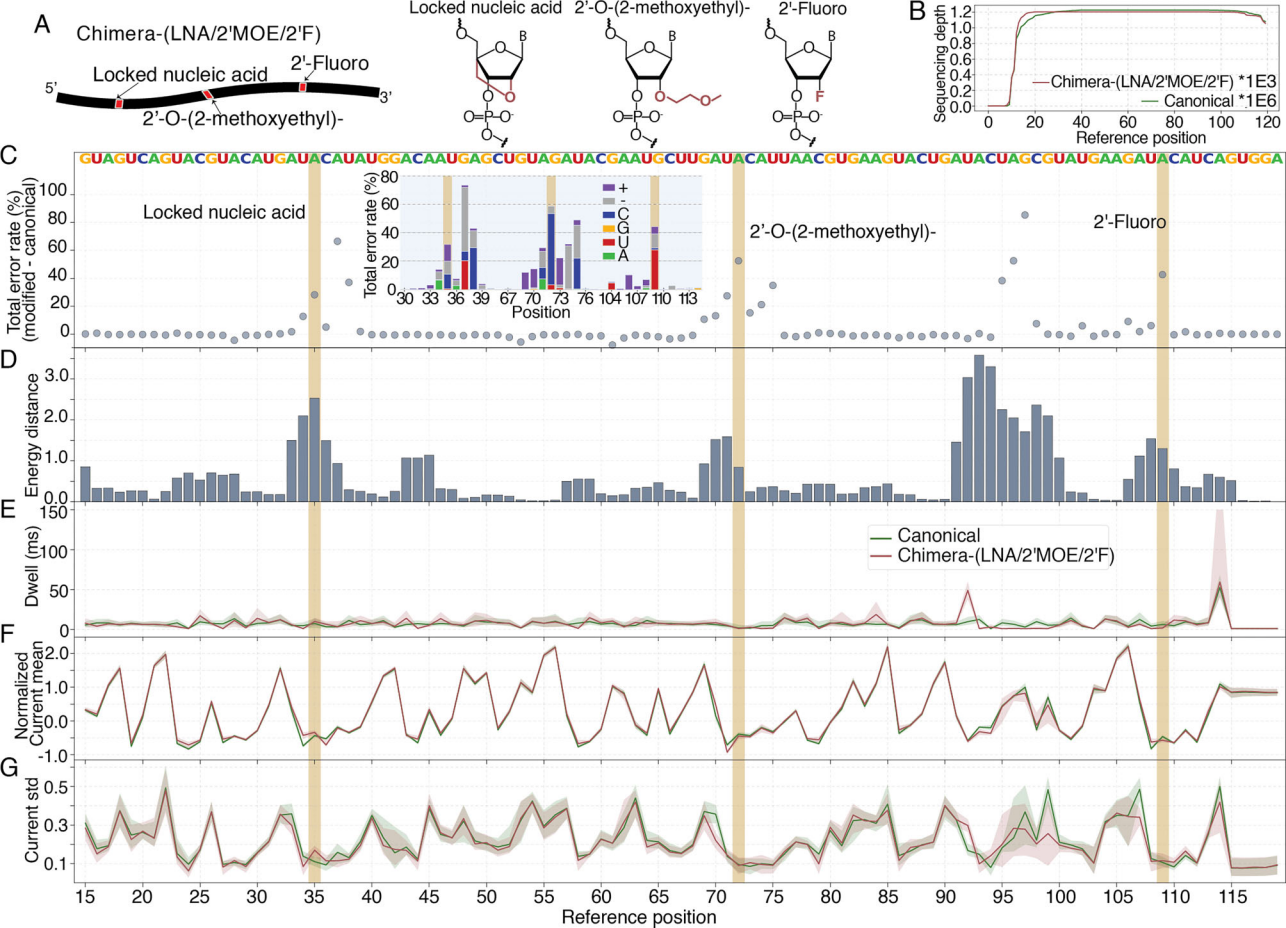
Error and current signatures of RNA modifications in Chimera-(LNA/2′MOE/2′F).(**A**) Schematic of Chimera-(LNA/2′MOE/2′F) showing LNA, 2′MOE, and 2′F modifications. Chemical structures are shown alongside. (**B**) Sequencing depth of canonical and modified RNA. Canonical RNA has ~1.2 × 10⁶ reads, while Chimera-(LNA/2′MOE/2′F) has ~1.2 × 10³. Both show decreased coverage at the 5′ end due to sequencing directionality and motor protein limitations. (**C**) Total error rate difference between Chimera-(LNA/2′MOE/2′F) and canonical RNA. Elevated total error rates are observed at and near modification sites. The inset shows a stacked box plot of the total error rate, with each stack representing a different type of error percentage. (**D**) Energy distance per position between canonical and Chimera-(LNA/2′MOE/2′F). Breakdown of current-level metrics: (**E**) Dwell time, (**F**) Normalized current mean, and (**G**) Current standard deviation. In all dwell time, current mean, and current standard deviation plots, the solid line represents the median across all reads, with the shaded area indicating the 25th to 75th percentile range. The highlighted regions indicate the modification sites, and the letters above the total error rate plots represent the reference sequence.

We next evaluated a construct [Chimera-(LNA/2′MOE/2′F)] incorporating three distinct modifications, each on an adenosine base: an LNA at position 35, a 2′MOE at position 72, and a 2′F at position 109 (Fig. 3A). Sequencing of Chimera-(LNA/2′MOE/2′F) (Fig. 3B) yielded ~1.2 × 10³ reads, a depth comparable to the Chimera-(PS/2′OMe) construct (Fig. 2B). All three sugar modifications, LNA, 2′MOE, and 2′F, contributed to a significant increase in the total error rate percentage, indicating that they each disrupt basecalling accuracy (Fig. 3C). LNA and 2′MOE not only affect the modification sites but also nearby bases. For the sequence context investigated here, they appear to preferentially cause C misscall errors, insertions, deletions, and U misscall errors (Fig. 3C inset).

Positions 95 to 98 produced a large total error rate (Fig. 3C), which was also reflected in the energy distance (Fig. 3D), where a rise is observed from positions 91 to 100. Breakdown of current into dwell (Fig. 3E), current mean (Fig. 3F), and current standard deviation (Fig. 3G) shows that there are indeed large differences between canonical and Chimera-(LNA/2′MOE/2′F) for all three metrics. Given the 3′-to-5′ direction of nanopore direct RNA sequencing, this current disturbance may be attributable to the 2′MOE modification at position 72. This suggests that the 2′MOE modification might induce a long-range signal disruption as far as 20 nt downstream, whereas the 2′OMe modification led to an increased dwell time over a shorter, ~10 nt range. Although the precise biophysical mechanisms remain to be characterized, 2′MOE induces complex signal distortions affecting both dwell time and current stability beyond simple translocation stalls. Consequently, future detection algorithms may account for these complexities, as bulky modifications might induce long-range signal effects extending beyond the immediate modification site.

The LNA modification at position 35 did not cause as pronounced a dwell time increase downstream as observed with 2′OMe. This difference may stem from the number of modifications; the helicase encounters only a single LNA, which it may overcome more quickly than the cluster of three 2′OMe modifications in the Chimera-(PS/2′OMe). While its effect on dwell time was subtle, the LNA has a larger impact on current mean and current standard deviation. LNA features a covalent bridge that “locks” the sugar into a fixed C3′-endo conformation. [48, 49] We speculate that structural rigidity shifts base positioning within the pore, causing distinct changes in mean current. At position 109, the 2′F modification induces only minor shifts in dwell time, current mean, and standard deviation, yet their cumulative effect drives a distinct increase in energy distance. This finding illustrates how multivariate approaches can detect modifications that might be overlooked by univariate inspection, such as dwell time analysis alone. To our knowledge, this study provides the first nanopore characterization of these therapeutic chemistries. These data offer a resource for future studies on therapeutic RNA and will aid in the development of robust sequencing algorithms.

IV. Assessing fully substituted base modifications

While sugar and backbone modifications such as 2′OMe, 2′MOE, LNA, 2′F, and PS are predominant in therapeutic gRNAs and siRNAs, therapeutic mRNAs rely heavily on internal base modifications to ensure safety and efficacy. We examined m1Ψ and 5moU, which are widely utilized to minimize immune stimulation [40]. We also investigated 5iodoC, a bulky modification often used to probe RNA–protein interactions, to further challenge the nanopore’s ability to resolve atypical modifications with heavy atoms [50]. Overall, 5moU modification produced higher sequencing depth compared to m1Ψ and 5iodoC, likely reflecting differences in compatibility with the motor protein and nanopore (Fig. S3). For all m1Ψ sequences and 5moU sequences (mCherry and eGFP) and 5iodoC mCherry sequences, the highest sequencing depth exceeds 60 000, 70 000, and 40 000, respectively. 5iodoC-modified eGFP has only up to 321 reads, most likely because eGFP has more C bases (240 Cs versus 180 Cs for mCherry). The higher number of 5iodoC affects how the motor protein and nanopore handle the modification, and the increased error rate associated with 5moU hinders read alignment to the reference.

**Figure 4.**
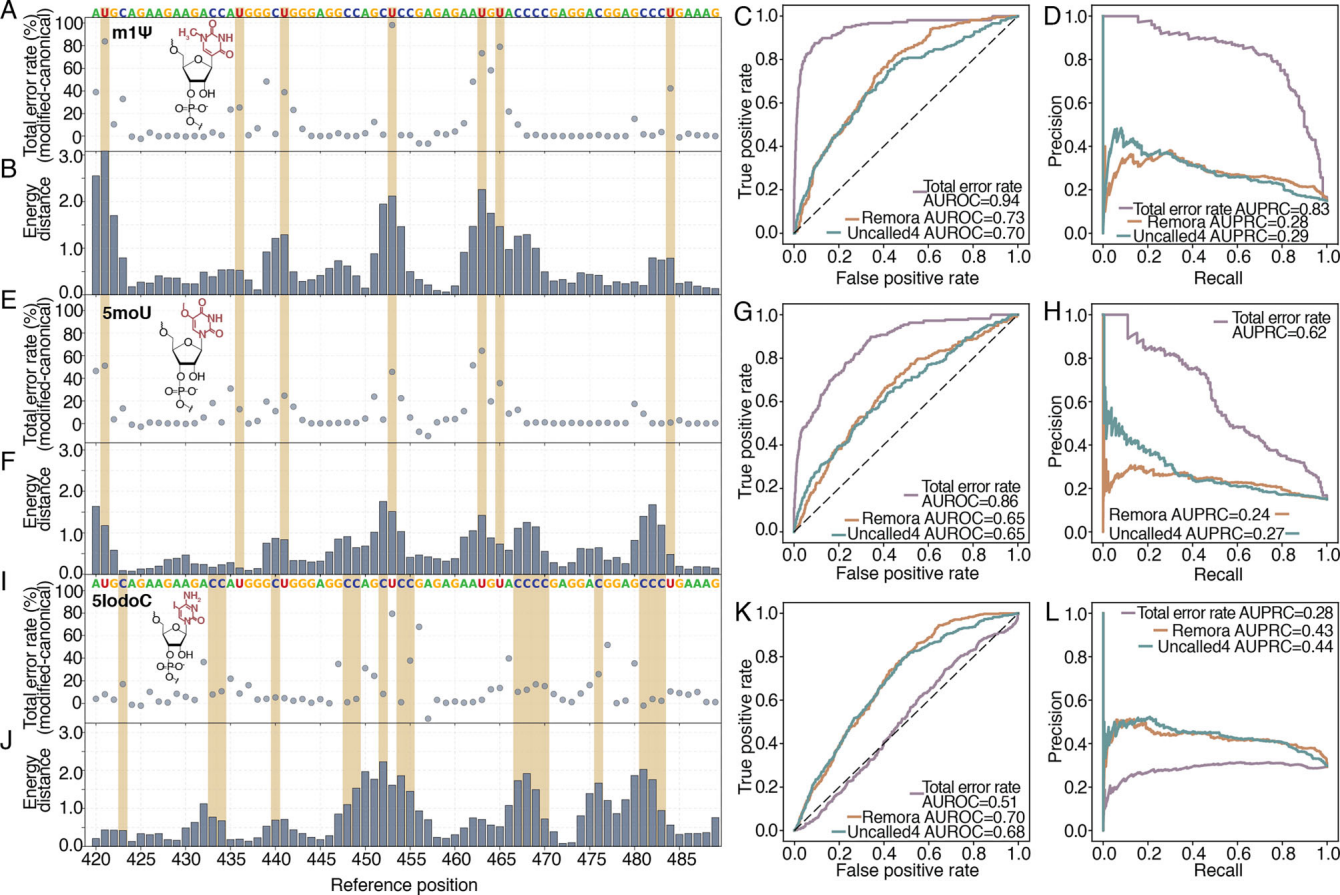
Base modification effects on total error rate and energy distance in mCherry and eGFP RNAs. (**A**) Total error rate for m1Ψ modification across a 70-nt mCherry region, showing increased error at and near modification sites. (**B**) Energy distance for m1Ψ, with peaks generally near modified positions, though some are less distinct. AUROC (**C**) and AUPRC (**D**) comparing total error rate and energy distance (Remora and Uncalled4 current extraction) for m1Ψ, using combined mCherry and eGFP data. (**E**) Total error rate for 5moU and (**F**) energy distance for 5moU. AUROC (**G**) and AUPRC (**H**) for 5moU, comparing total error rate and energy distance metrics. (**I**) Total error rate for 5iodoC and (**J**) energy distance for 5iodoC. AUROC (**K**) and AUPRC (**L**) for 5iodoC. The highlighted regions indicate the modification sites, and the letters above the total error rate plots represent the reference sequence.

The canonical control was sequenced with high accuracy, as the total error rate remained below 10% for most nucleotide positions and only rarely exceeded 20% (Supplementary Fig. S4). For all sequences examined, regardless of modification, clear peaks and patterns are less obvious compared with Chimera-(PS/2′OMe). This is due to 100% substitution of U with m1Ψ or 5moU, or 100% substitution of C with 5iodoC, causing the ionic signals of individual modifications to merge and consequently making single-modification resolution difficult. For m1Ψ, the overall total error rate and energy distance increase at modification and adjacent sites (Fig. 4A), though its prominence of energy distance was observed to vary (e.g. position 436 in Fig. 4B). A comparable trend is observed for 5moU, which generally had a lower total error rate compared to m1Ψ (Fig. 4E and F). The mean and median total error rates at m1Ψ modified positions were 63.80% and 72.20%, respectively. For 5moU, these values were 40.83% and 37.67%, respectively. The comparatively lower total error rate for 5moU may be attributed to its structural similarity to canonical U, differing only by the addition of a methoxy group at the 5th carbon of the uridine base. Conversely, in m1Ψ, the uracil base is attached to the ribose sugar via a C-glycosidic bond (C5-C1′) rather than an N-glycosidic bond, and an additional methyl group is present at the N1 position. When examining the total error rate, both m1Ψ and 5moU are preferentially misidentified as C or deletions (Supplementary Fig. S5), consistent with prior observations for Ψ or m1Ψ using earlier versions of the direct RNA ONT chemistry [51]. The 5iodoC dataset is more complex than m1Ψ and 5moU. While the error rate at modified positions is moderate (mean 16.47%, median 11.13%), the surrounding bases exhibit elevated errors (Fig. 4I). Nevertheless, energy distance metrics successfully detect these sites, confirming the signal persists despite basecalling challenges (Fig. 4J).

Besides energy distance with the current matrix extracted using Remora, we also extracted the current matrix using Uncalled4. Remora employs a dynamic programming algorithm to segment the raw signal against the sequence of k-mers from the basecalled read, whereas Uncalled4 uses a move-table-guided dynamic time warping algorithm to align the signal against the k-mer sequence from a known reference. Consequently, for detecting RNA modifications, Uncalled4 offers robust alignment to the reference at the risk of normalizing the distinguishing signal features, whereas Remora preserves the raw signal at the risk of misalignment from basecalling errors. In general, the energy distance peaks still approximately align with the total error rate peaks similar to Remora (Supplementary Figs S6–S8).

To evaluate the performance of each metric in distinguishing modified bases, we computed the area under the receiver operating characteristic curve (AUROC) and area under the precision–recall curve (AUPRC) for the total error rate and energy distance matrices derived from Remora and Uncalled4 (Fig. 4C and D for m1Ψ, Fig. 4G and H for 5moU, and Fig. 4K and L for 5iodoC). All AUROC and AUPRC values are calculated using combined data from mCherry and eGFP. For both m1Ψ and 5moU, the total error rate outperformed energy distance, proving relatively effective for m1Ψ detection with an AUROC of 0.94 and AUPRC of 0.83. This is expected since energy distance affects a broader region of nearby bases, as seen in Chimera-(PS/2′OMe). To better understand performance, if we use a 20% total error rate threshold, then we obtain 101 true positives, 97 false positives, 700 true negatives, and 13 false negatives for m1Ψ mCherry. Among the 97 false positives, 96 can be explained by being within a 7 nt k-mer window of true m1Ψ modifications. Conversely, for 5iodoC, the total error rate performed ineffectively. In this case, the energy distance metric of 5iodoC from both Remora and Uncalled4 matrices yielded AUROC and AUPRC values similar to those observed for m1Ψ and 5moU. Overall, our results indicate that current nanopore applications for therapeutic mRNA face challenges with full substitution of modifications, as the ionic signals of individual modifications tend to merge and overlap. While the total error rate offers a promising avenue for detecting m1Ψ and 5moU, accurate detection currently relies on prior knowledge of the specific modification type present.

V. Benchmarking modification-aware basecalling

2′OMe is employed in therapeutic guide RNAs and siRNA to enhance stability and suppress immune activation, making the verification of modification stoichiometry a critical quality control step. While recent benchmarks [45] assessed RNA004 on biological samples with variable modification levels, our study targets the demands of therapeutic product evaluation. By utilizing pure synthetic constructs with defined 100% stoichiometry, this study aims to assess the quantitative performance and basecalling errors.

To this end, we evaluated Dorado’s modification-aware models on Chimera-(PS/2′OMe), Cluster-2′OMePS, and m5C mCherry and eGFP sequences. To mimic the clustered modification landscapes typical of therapeutic guide RNAs and evaluate the impact of modification density on detection, Cluster-2′OMePS contains consecutive one (position 109), two (positions 71, 72), and three (positions 34, 35, 36) 2′OMePS modifications with different base contexts. Because current 2′OMe basecalling models are base-specific, we applied the adenosine model to Chimera-(PS/2′OMe) and the adenosine, cytidine, and uridine models to Cluster-2′OMePS. Modification percentages were subsequently quantified using Modkit. The canonical control reached up to 1 559 400 sequencing depth, Chimera-(PS/2′OMe) up to 42 718, and Cluster-2′OMePS up to 949 493 (Supplementary Fig. S9).

**Figure 5.**
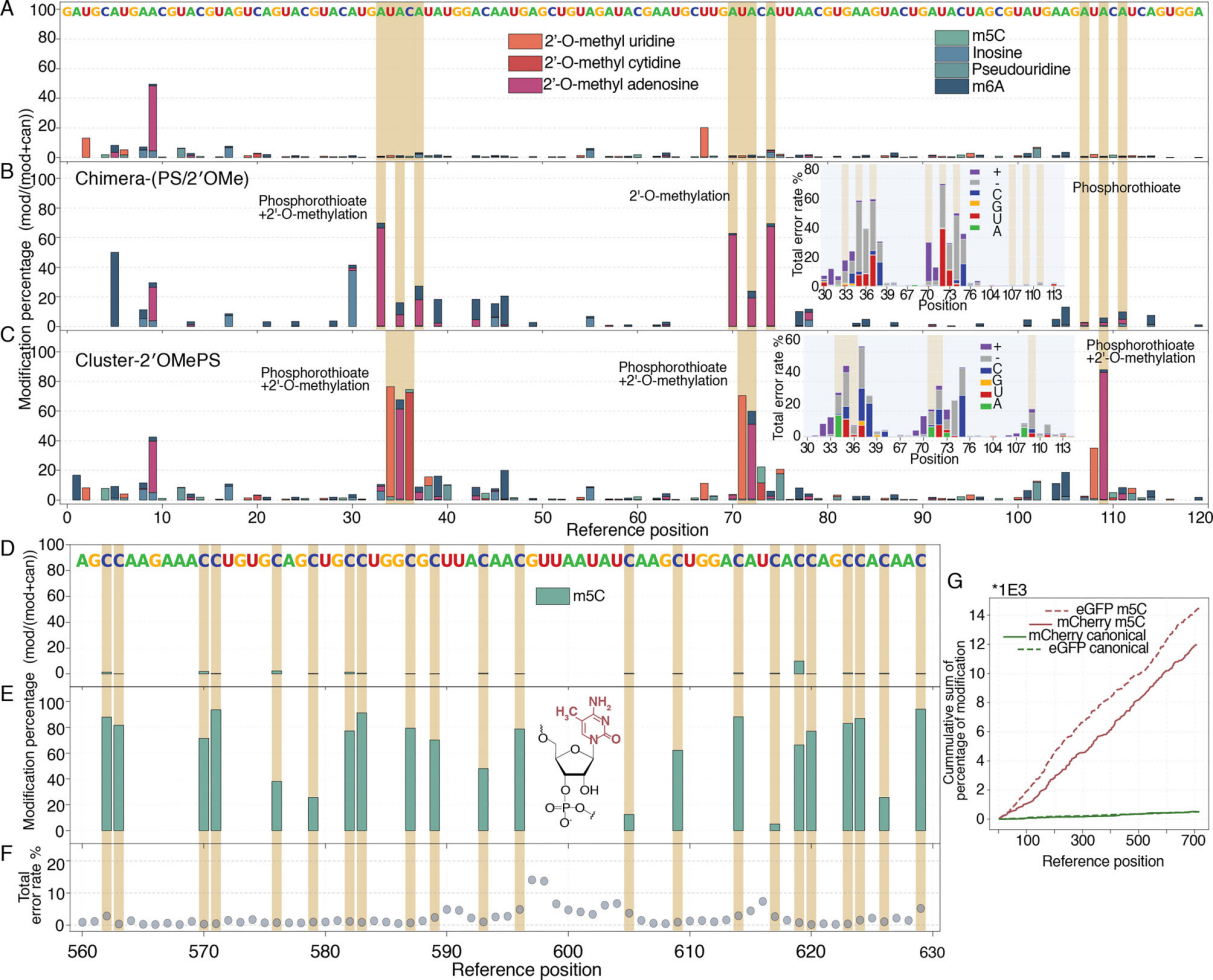
Detection of RNA modifications using modification-aware basecalling models. (**A**) Modification percentage across the canonical control RNA, showing low background. (**B, C**) Chimera-(PS/2′OMe) and Cluster-2′OMePS modification calls. The basecaller correctly identifies 2′OMe modifications, distinguishing them from other types, but quantification is incomplete, with underestimation sites. Insets show a stacked box plot of total error rates. (**D**) Modification calls for canonical mCherry and (**E**) m5C-modified mCherry RNA using the m5C-aware basecaller, with increased calls at modified sites but incomplete quantification. (**F**) Representative total error rates for m5C-modified mCherry, highlighting lower error rates for m5C-aware basecaller compared to 2′OMe. (**G**) Cumulative modification percentage for canonical and m5C-modified mCherry and eGFP transcripts, showing an increase along the transcript compared to flat profiles in canonical controls.

To evaluate performance, we first assessed the basecalling accuracy. Analysis of the 2′OMe-aware basecalling models revealed high error rates at modified sites. For instance, at position 34 of Cluster-2′OMePS, approximately 27.2% of reads were erroneous, comprising 11.0% deletions, 13.5% A-miscalls, 1.6% insertions, and minor C/G miscalls (Fig. 5C—Inset). Consequently, the reported 2′OMe-uridine modification percentage (74.3%) is derived only from the remaining ~73% of reads that were correctly basecalled as uridine. This elevated error profile is driven largely by deletions and miscalls and persists whether the modification is 2′OMe alone (positions 70, 72, 74) or combined 2′OMePS. In comparison, the m5C-aware basecalling models demonstrated superior basecalling accuracy. The total error rate for m5C-modified mCherry was substantially lower than that observed for 2′OMe, with total variance remaining below 20% at most positions (Fig. 5F and Supplementary Fig. S10). Similarly, the combined 2′OMe-cytidine/m5C model maintained error rates comparable to the m5C-only model (Supplementary Fig. S11). However, for m1Ψ, the pseudouridine-aware model yielded elevated error rates, likely because the single methyl group difference creates current variations that the model, trained on standard pseudouridine, struggles to resolve (Supplementary Fig. S12).

Despite the error profiles described earlier, the modification-aware basecalling model successfully detected the presence of modifications, though quantitative limitations remain. As anticipated, canonical control RNAs exhibited low background modification frequencies (Fig. 5A and D), with only minor artifacts such as a peak at position 9 attributable to motor protein instability near the 5′ end. For the synthetic constructs, the basecalling models correctly distinguished 2′OMe from other modifications (m6A, inosine, pseudouridine, m5C) and successfully identified m5C sites in mCherry/eGFP (Fig. 5B and C, E). However, the measured modification percentages consistently failed to reach the expected 100% stoichiometry inherent to these synthetic standards. For 2′OMe, we observed modification calls increased in regions immediately flanking true sites (e.g. positions 37–40 for Cluster-2′OMePS). Similarly, m5C quantification was significantly underestimated at several positions; notably, position 617 registered <10% modification despite being a 100% modified site. Sequence context appears to have influence, as a uracil immediately preceding an m5C site correlated with lower detection rates (Supplementary Fig. S13). The combined 2′OMe-cytidine/m5C model correctly identified the majority of m5C sites but showed specific instances (e.g. position 401) where m5C was misidentified as 2′OMe-cytidine (Supplementary Fig. S14). These trends are summarized in Fig. 5G, where the cumulative sum of modification percentage increases steadily for modified mRNAs while remaining flat for controls, confirming detection capability despite the quantitative underestimation.

### Discussion

This study systematically benchmarked Oxford Nanopore’s latest direct RNA sequencing chemistry (SQK-RNA004) for detecting a diverse range of modifications. Our findings establish that this technology is a potentially powerful tool with direct relevance for biomanufacturing and quality control of therapeutic RNAs, especially mRNAs. A key advantage over conventional methods like HPLC/MS is its ability to deliver multi-kilobase read lengths, which is critical for full-length transcript characterization and the potential detection of sequence variants in therapeutic mRNA [52]. It holds potential for *de novo* sequencing, which is not feasible for MS-based methods that rely on a reference.

To our knowledge, this is the first time that some of these modifications have been explored, underscoring the necessity for developing comprehensive modification-aware basecallers. For modifications currently lacking dedicated models, we found that total error rate analysis is generally sufficient for detecting structurally pronounced sugar (e.g. 2′OMe, 2′MOE, LNA) and base (e.g. m1Ψ, 5moU) modifications. However, this metric is ineffective for PS backbone modifications, which induce minimal basecalling errors; detecting these subtle modifications instead requires raw signal analysis, such as energy distance. Additionally, our benchmarking of released modification-aware basecallers (2′OMe, m5C) revealed that while they successfully identify targets, they consistently underestimate modification stoichiometry. Notably, the m5C basecaller exhibited a substantially lower error rate than the 2′OMe model, highlighting variability in current tool performance.

It is important to acknowledge the experimental constraints of this study. To ensure higher reliability, we relied on synthetic oligonucleotides restricted to ~120 nt due to the high cost and complexity of chemical synthesis. This length is directly relevant for therapeutic classes such as CRISPR gRNAs (typically ~100 nt), however, it limits the sequence space available to explore diverse sequence contexts or complex combinations of modifications. Additionally, the scope of our benchmarking was bounded by the commercial availability of therapeutically relevant modifications. Despite our focus on internal modifications, we noted reduced terminal coverage caused by unregulated translocation speeds at the strand ends. For future terminal analysis, such as 5′-caps or gRNA terminal modifications, ligating flanking RNA adaptors can “shield” these regions, shifting them into the reading window [53, 54]. Furthermore, terminal modifications often inhibit standard poly(A) polymerase enzymes. To address this, specialized workflows are needed to employ custom reverse transcription adapters (RTAs) that bind directly to the 3′ end [53, 54], or utilize engineered poly(A) polymerases and RNA ligases compatible with 3′-modified termini. Consequently, while we demonstrated the feasibility of detecting these chemistries, future efforts must expand into broader sequence contexts to fully validate performance for longer transcripts.

In summary, this work provides an exploratory assessment of direct RNA sequencing for native and therapeutic RNA modifications. Despite current limitations in quantification and tool availability, it establishes analytical performance baselines for enabling nucleic acid-based therapeutic development and interpreting future naturally occurring biological modifications, demonstrated here through the characterization of simple, synthetic variants with defined stoichiometry.

## Supplementary Material

gkag411_Supplemental_File

## Data Availability

The sequencing data generated in this study have been deposited in the Sequence Read Archive (SRA) under accession number PRJNA1404165. All other data supporting the findings of this study are available within the article and its supplementary materials.
