# Supplementary material for: Systematic assessment of diverse RNA modifications using nanopore direct RNA sequencing
Source: Nucleic Acids Res. 2026 May 4;54(8):gkag411. doi: 10.1093/nar/gkag411 (PMC13136890; doi:10.1093/nar/gkag411)
Supplement: gkag411_Supplemental_File [file gkag411_supplemental_file.docx]

#

Supporting information

# Systematic Assessment of Diverse RNA Modifications Using Nanopore Direct RNA Sequencing

Xinqi Kang^1^, Kelly Zhang^1^, Alexandre Goyon^1^*, William Stephenson^2^*

^1^Department of Synthetic Molecule Analytical Chemistry, Genentech, 1 DNA Way, South San Francisco, California 94080, United States

^2^Department of Proteomic and Genomic Technologies, Genentech, 1 DNA Way, South San Francisco, California 94080, United States

Email: stephew3@gene.com

Email: [goyona@gene.com](mailto:goyona@gene.com)

## Supplementary Text S1: Sequence and description of custom RNA constructs

1. Site-specifically modified 120-nt synthetic RNAs
   1. Canonical

rGrArUrGrCrArUrGrArArCrGrUrArCrGrUrArGrUrCrArGrUrArCrGrUrArCrArUrGrArUrArCrArUrArUrGrGrArCrArArUrGrArGrCrUrGrUrArGrArUrArCrGrArArUrGrCrUrUrGrArUrArCrArUrUrArArCrGrUrGrArArGrUrArCrUrGrArUrArCrUrArGrCrGrUrArUrGrArArGrArUrArCrArUrCrArGrUrGrGrA

- 1. Chimera-(PS/2′OMe)

rGrArUrGrCrArUrGrArArCrGrUrArCrGrUrArGrUrCrArGrUrArCrGrUrArCrArUrGmA*rUmA*rCmA*rUrArUrGrGrArCrArArUrGrArGrCrUrGrUrArGrArUrArCrGrArArUrGrCrUrUrGmArUmArCmArUrUrArArCrGrUrGrArArGrUrArCrUrGrArUrArCrUrArGrCrGrUrArUrGrArArGrA*rUrA*rCrA*rUrCrArGrUrGrGrA

- 1. Chimera-(LNA/2′MOE/2′F)

rGrArUrGrCrArUrGrArArCrGrUrArCrGrUrArGrUrCrArGrUrArCrGrUrArCrArUrGrArU+ArCrArUrArUrGrGrArCrArArUrGrArGrCrUrGrUrArGrArUrArCrGrArArUrGrCrUrUrGrArU/i2MOErA/rCrArUrUrArArCrGrUrGrArArGrUrArCrUrGrArUrArCrUrArGrCrGrUrArUrGrArArGrArU/i2FA/rCrArUrCrArGrUrGrGrA

- 1. Cluster-2′OMePS

rGrArUrGrCrArUrGrArArCrGrUrArCrGrUrArGrUrCrArGrUrArCrGrUrArCrArUrGrAmU*mA*mC*rArUrArUrGrGrArCrArArUrGrArGrCrUrGrUrArGrArUrArCrGrArArUrGrCrUrUrGrAmU*mA*rCrArUrUrArArCrGrUrGrArArGrUrArCrUrGrArUrArCrUrArGrCrGrUrArUrGrArArGrArUmA*rC rArUrCrArGrUrGrGrA

1. IVT mRNAs:

The mCherry and eGFP mRNA constructs were purchased from GenScript. The specific sequences of the 5′ and 3′ untranslated regions (UTRs) are proprietary to the manufacturer and were not disclosed.

Modified constructs were synthesized with a complete substitution of corresponding canonical bases: N1MeΨ and 5moU fully replace U, and 5iodoC and m5C fully replace C.

- 1. mCherry

5'UTR+Kozak+AUGCUGUCUAAGGGCGAGGAAGAUAACAUGGCCAUCAUCAAGGAAUUCAUGAGAUUCAAGGUCCACAUGGAAGGUUCUGUGAAUGGCCAUGAGUUUGAGAUCGAGGGAGAAGGCGAAGGAAGACCUUACGAGGGCACACAGACCGCCAAGCUGAAGGUGACAAAGGGCGGCCCUCUGCCCUUCGCCUGGGAUAUCCUGUCCCCUCAGUUCAUGUACGGCAGCAAGGCCUAUGUGAAGCACCCUGCUGAUAUCCCCGACUACCUGAAGCUGAGCUUCCCAGAGGGCUUCAAGUGGGAAAGAGUGAUGAACUUCGAGGACGGCGGCGUGGUGACCGUGACCCAGGACAGCAGCCUGCAAGAUGGAGAAUUUAUCUACAAGGUGAAACUGCGGGGCACCAACUUCCCUAGCGACGGCCCCGUGAUGCAGAAGAAGACCAUGGGCUGGGAGGCCAGCUCCGAGAGAAUGUACCCCGAGGACGGAGCCCUGAAAGGCGAGAUUAAGCAGCGGCUGAAGCUCAAGGACGGCGGACACUACGACGCCGAGGUGAAAACCACCUACAAAGCCAAGAAACCUGUGCAGCUGCCUGGCGCUUACAACGUUAAUAUCAAGCUGGACAUCACCAGCCACAACGAGGAUUAUACAAUCGUGGAACAGUACGAGCGGGCCGAAGGCAGACACAGCACAGGCGGCAUGGACGAGCUGUACAAGUAA+3'UTR+100nt Poly(A)

- 1. eGFP

5'UTR+Kozak+AUGGUGAGCAAGGGCGAGGAGCUGUUCACCGGGGUGGUGCCCAUCCUGGUCGAGCUGGACGGCGACGUAAACGGCCACAAGUUCAGCGUGUCCGGCGAGGGCGAGGGCGAUGCCACCUACGGCAAGCUGACCCUGAAGUUCAUCUGCACCACCGGCAAGCUGCCCGUGCCCUGGCCCACCCUCGUGACCACCCUGACCUACGGCGUGCAGUGCUUCAGCCGCUACCCCGACCACAUGAAGCAGCACGACUUCUUCAAGUCCGCCAUGCCCGAAGGCUACGUCCAGGAGCGCACCAUCUUCUUCAAGGACGACGGCAACUACAAGACCCGCGCCGAGGUGAAGUUCGAGGGCGACACCCUGGUGAACCGCAUCGAGCUGAAGGGCAUCGACUUCAAGGAGGACGGCAACAUCCUGGGGCACAAGCUGGAGUACAACUACAACAGCCACAACGUCUAUAUCAUGGCCGACAAGCAGAAGAACGGCAUCAAGGUGAACUUCAAGAUCCGCCACAACAUCGAGGACGGCAGCGUGCAGCUCGCCGACCACUACCAGCAGAACACCCCCAUCGGCGACGGCCCCGUGCUGCUGCCCGACAACCACUACCUGAGCACCCAGUCCGCCCUGAGCAAAGACCCCAACGAGAAGCGCGAUCACAUGGUCCUGCUGGAGUUCGUGACCGCCGCCGGGAUCACUCUCGGCAUGGACGAGCUGUACAAGUAA+3'UTR+100nt Poly(A)

## Section 1. Poly(A) tailing baseline profiling of canonical site-specifically modified 120-nt synthetic RNAs


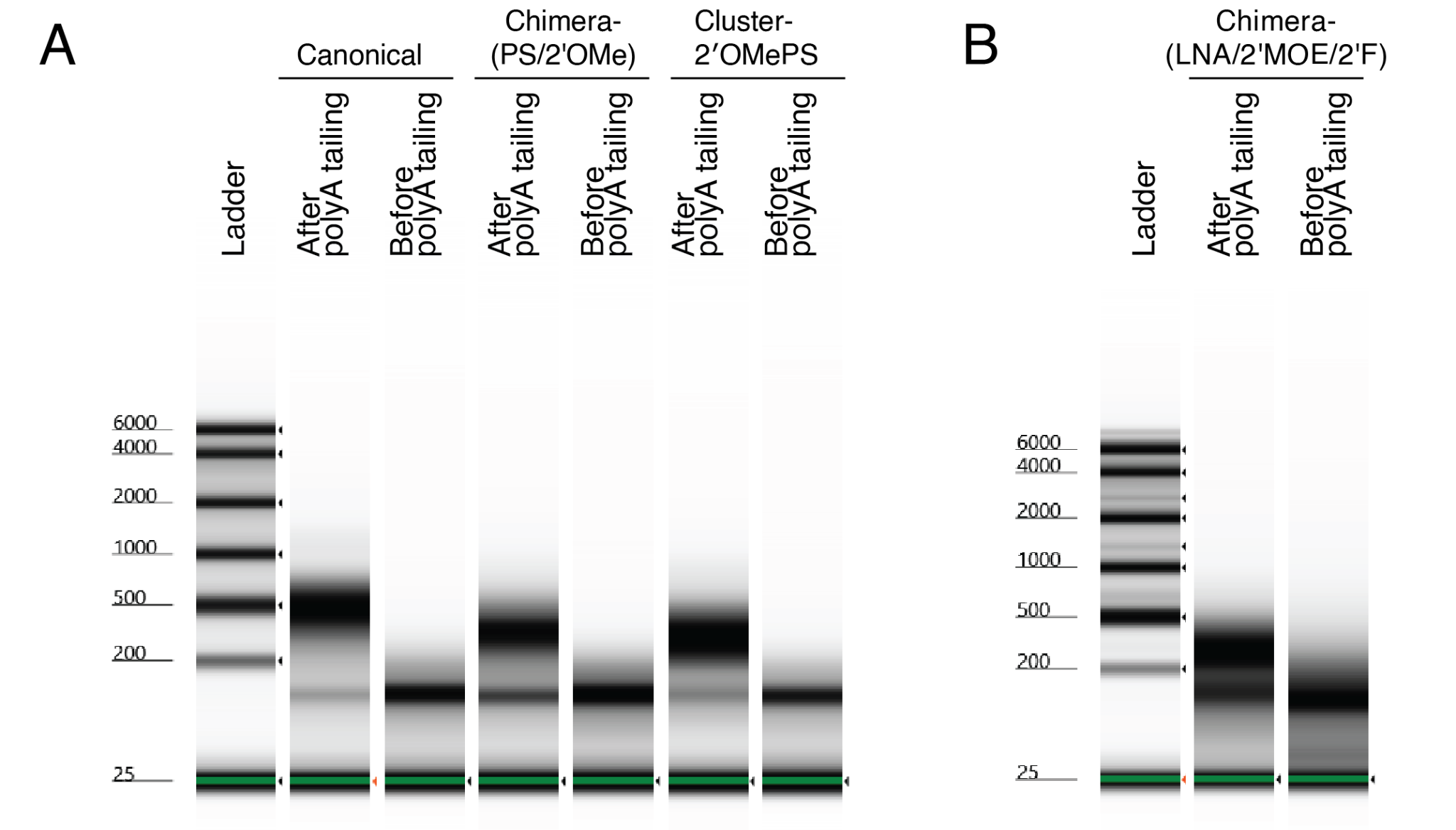


Figure S1. Poly(A) tailing of site-specifically modified 120-nt synthetic RNAs. (A) TapeStation image of Canonical, Chimera-(PS/2′OMe), and Cluster-2′OMePS RNAs before and after poly(A) tailing. (B) TapeStation image of Chimera-(LNA/2′MOE/2′F) before and after poly(A) tailing. Following poly(A) tailing, a shift in the observed RNA length indicates successful polyadenylation.


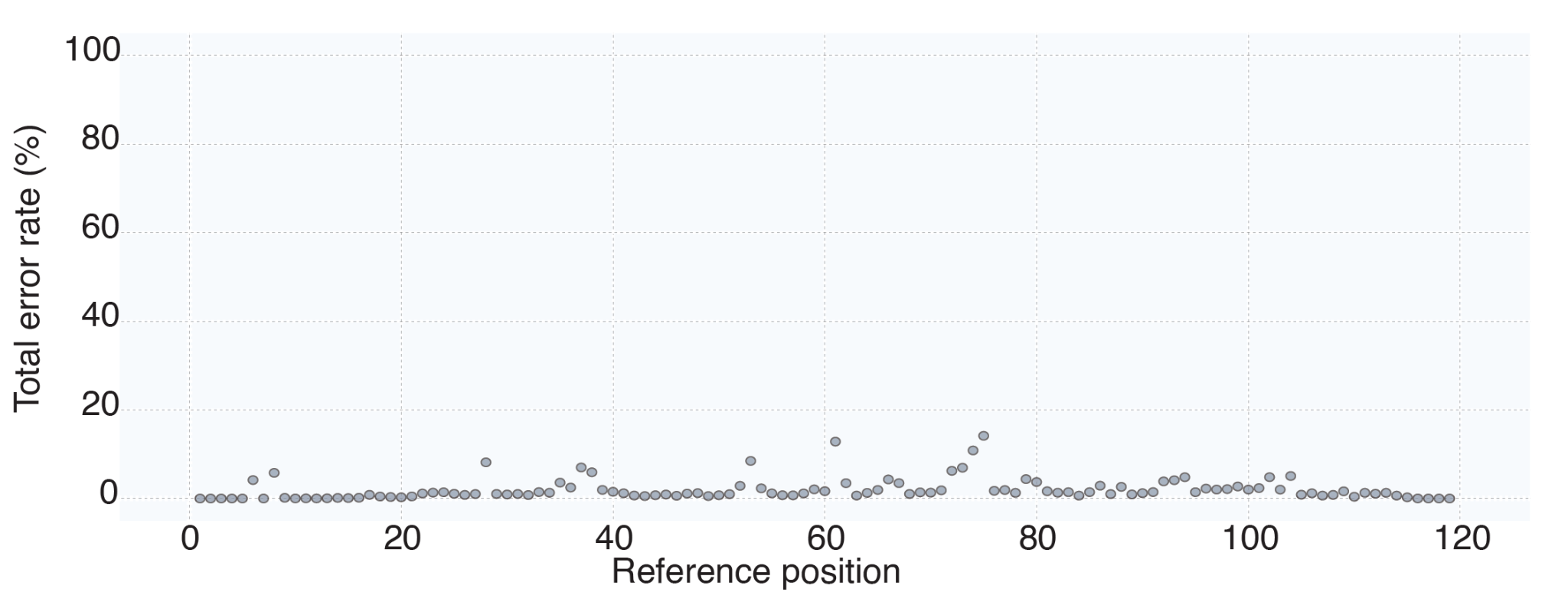


Figure S2. The total error rate of the canonical control of site-specifically modified 120-nt synthetic RNAs. Most of the canonical control is correctly called, with the total error rate under 10% in most cases and never exceeding 20%.

## Section 2. Sequencing statistics and signal signatures of IVT mRNAs


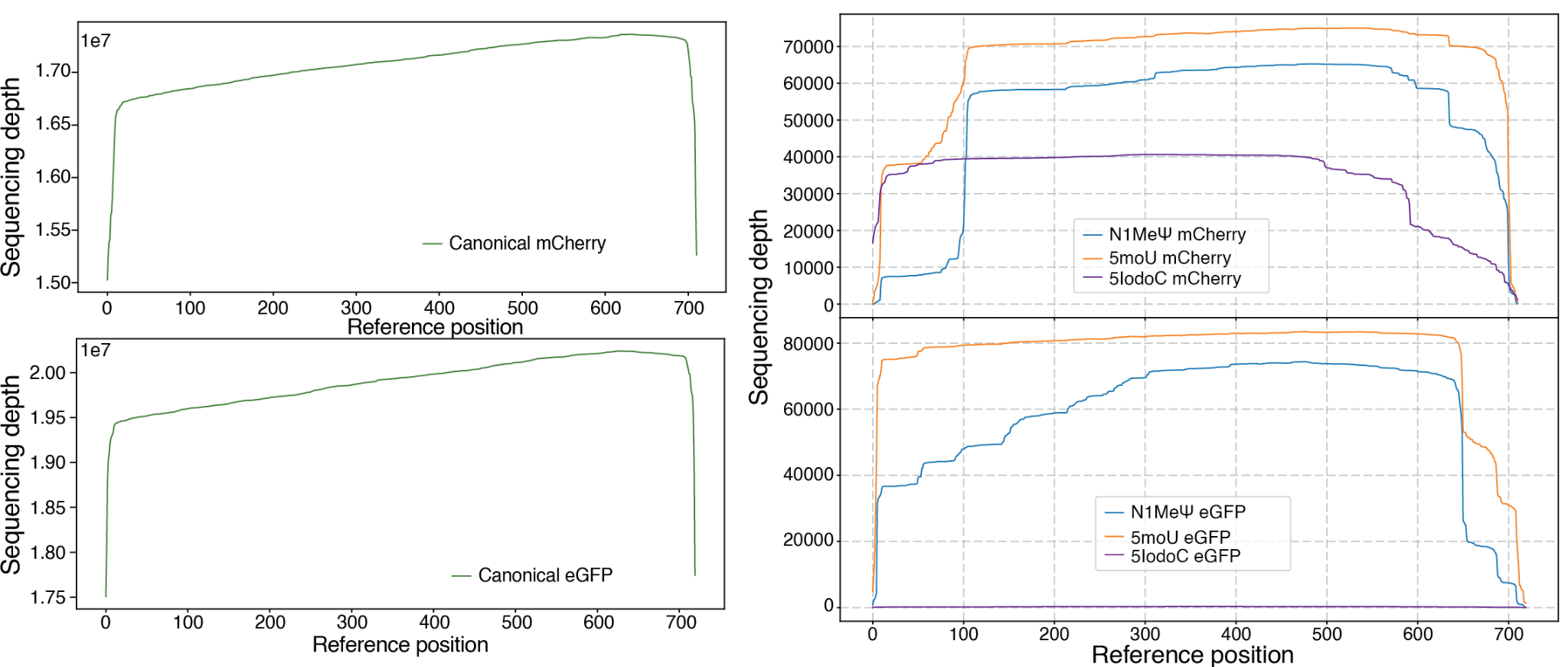


Figure S3. Sequencing depth of Canonical, N1MeΨ, 5moU, and 5iodoC mCherry and eGFP IVT mRNAs. For all N1MeΨ sequences (mCherry and eGFP), 5moU sequences (mCherry and eGFP), and 5iodoC mCherry sequences, the maximal sequencing depth exceeds 60,000, 70,000, and 40,000, respectively. 5iodoC-modified eGFP exhibits a maximum of 321 reads. Data are basecalled using rna004_130bps_hac@v3.0.1. Reads are filtered to exclude secondary and unmapped sequences, retaining only uniquely mapped reads with a minimum mapping quality of Q20.


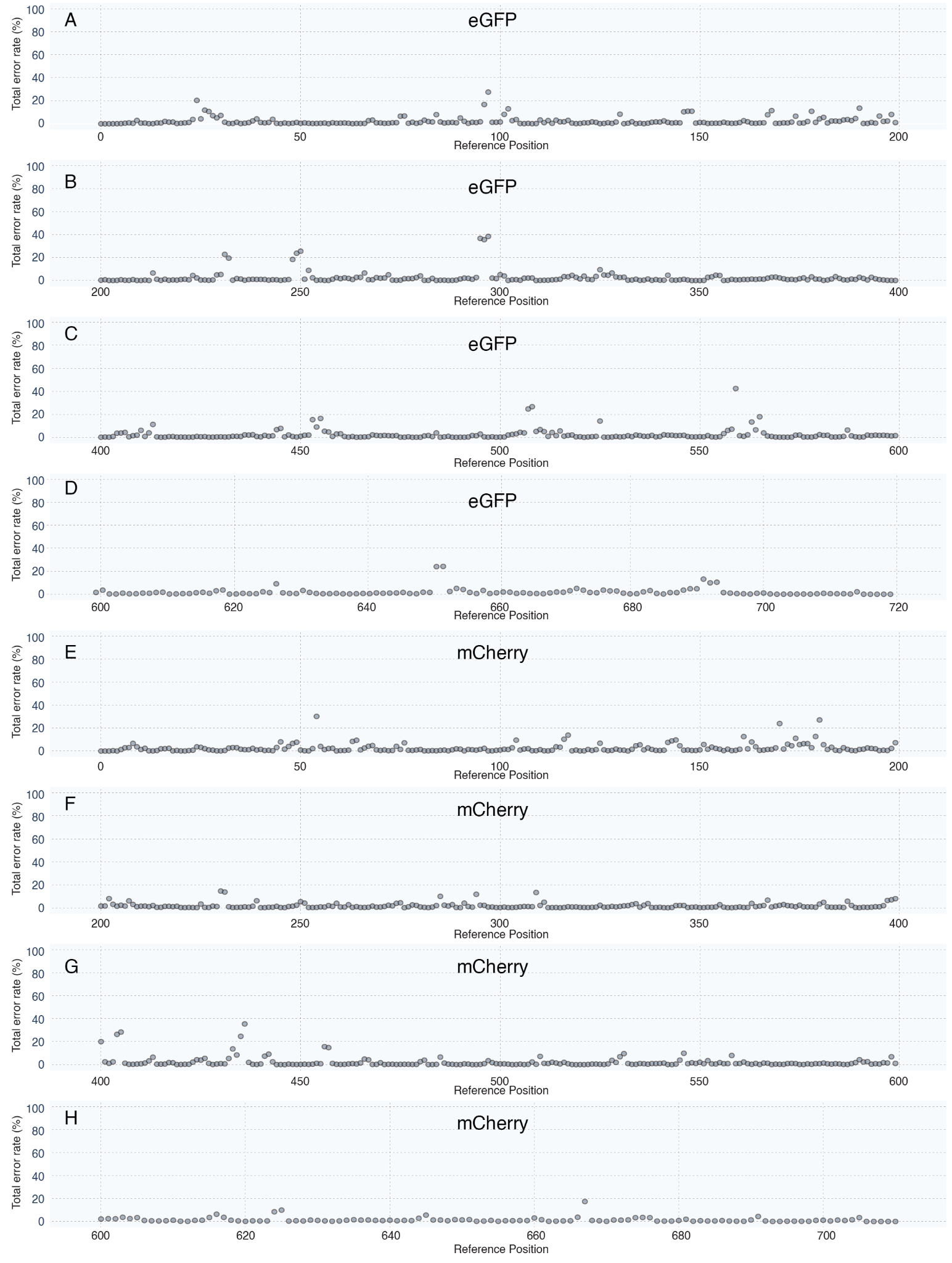


Figure S4. Total error rates of canonical mRNA controls. Error rates were calculated for mRNAs basecalled using rna004_130bps_hac@v3.0.1. (A–D) Total error rates for eGFP mRNAs. (E–H) Total error rates for mCherry mRNAs. For most positions, the canonical control is correctly identified, with total error rates generally remaining below 10% and only a few isolated positions exceeding 20%.


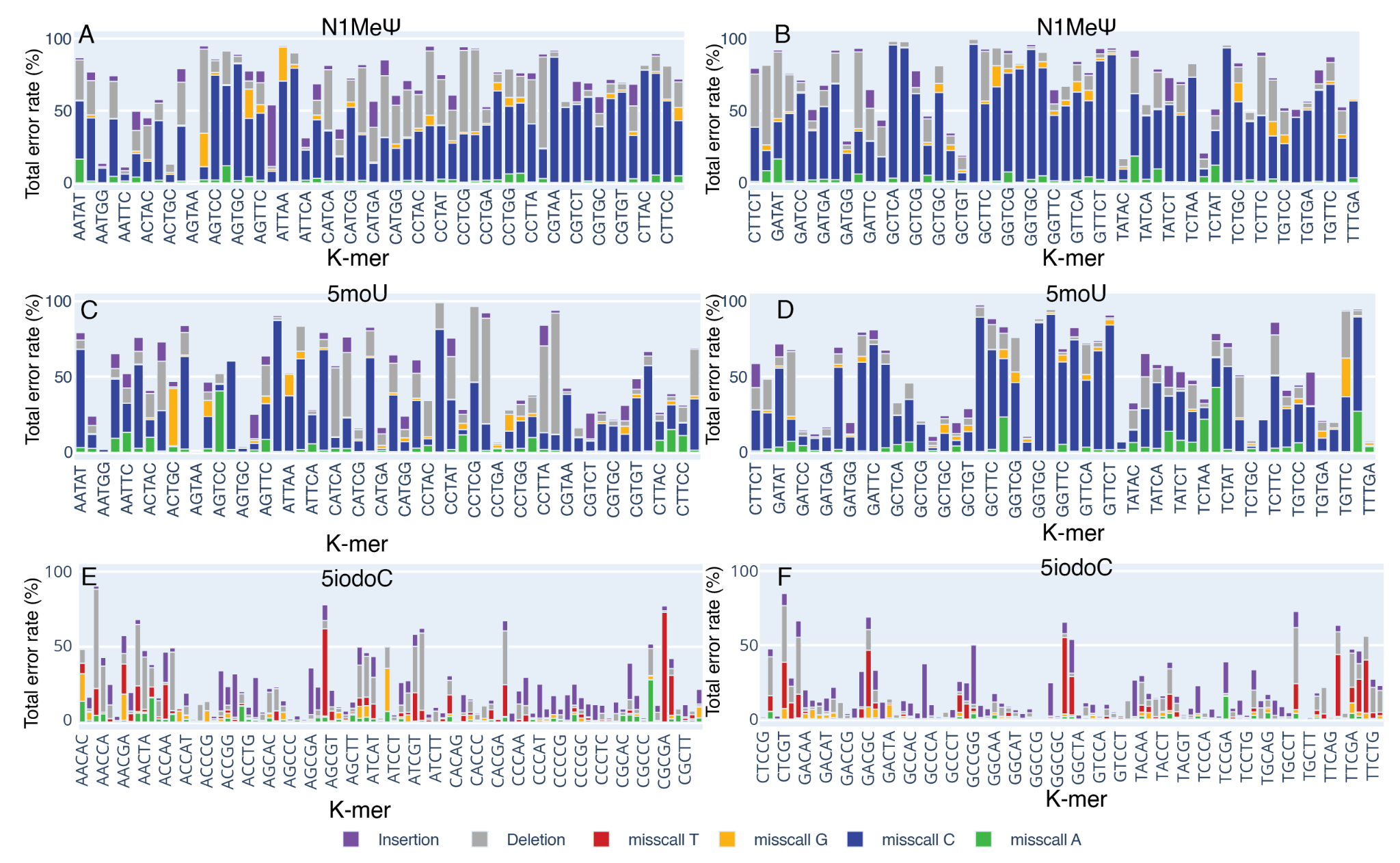


Figure S5. Stacked bar plots depicting the total error rate for each k-mer. (A-B) Stacked bar plot illustrating the total error rate of N1MeΨ 5-nucleotide k-mers. (C-D) Stacked bar plot illustrating the total error rate of 5moU 5-nucleotide k-mers. (E-F) Stacked bar plot illustrating the total error rate of 5iodoC 5-nucleotide k-mers. N1MeΨ and 5moU are preferentially identified as cytosine errors.


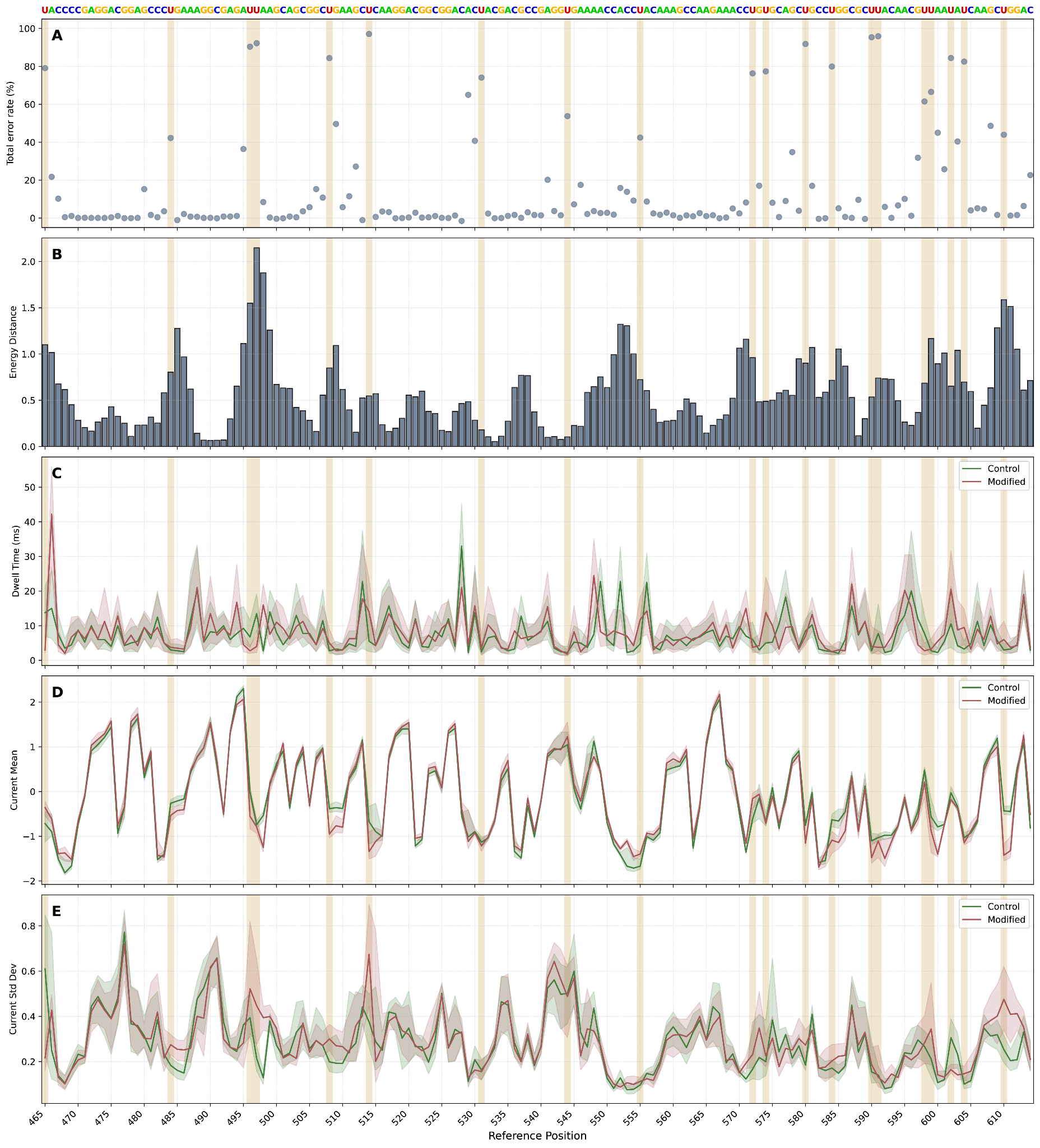


Figure S6. Error and current signatures of RNA modifications in N1MeΨmCherry mRNA from position 465-615. (A) Total error rate difference between N1MeΨmCherry mRNA and canonical mRNA. (B) Energy distance per position between N1MeΨmCherry mRNA and canonical mRNA. Breakdown of current-level metrics: (C) Dwell time (D) Normalized current mean (E) Current standard deviation. In all dwell time, current mean, and current standard deviation plots, the solid line represents the median across all reads, with the shaded area indicating the 25th to 75th percentile range. The highlighted regions indicate the modification sites, and the letters above the total error rate plots represent the reference sequence. Raw current signal alignment is performed by Uncalled4.


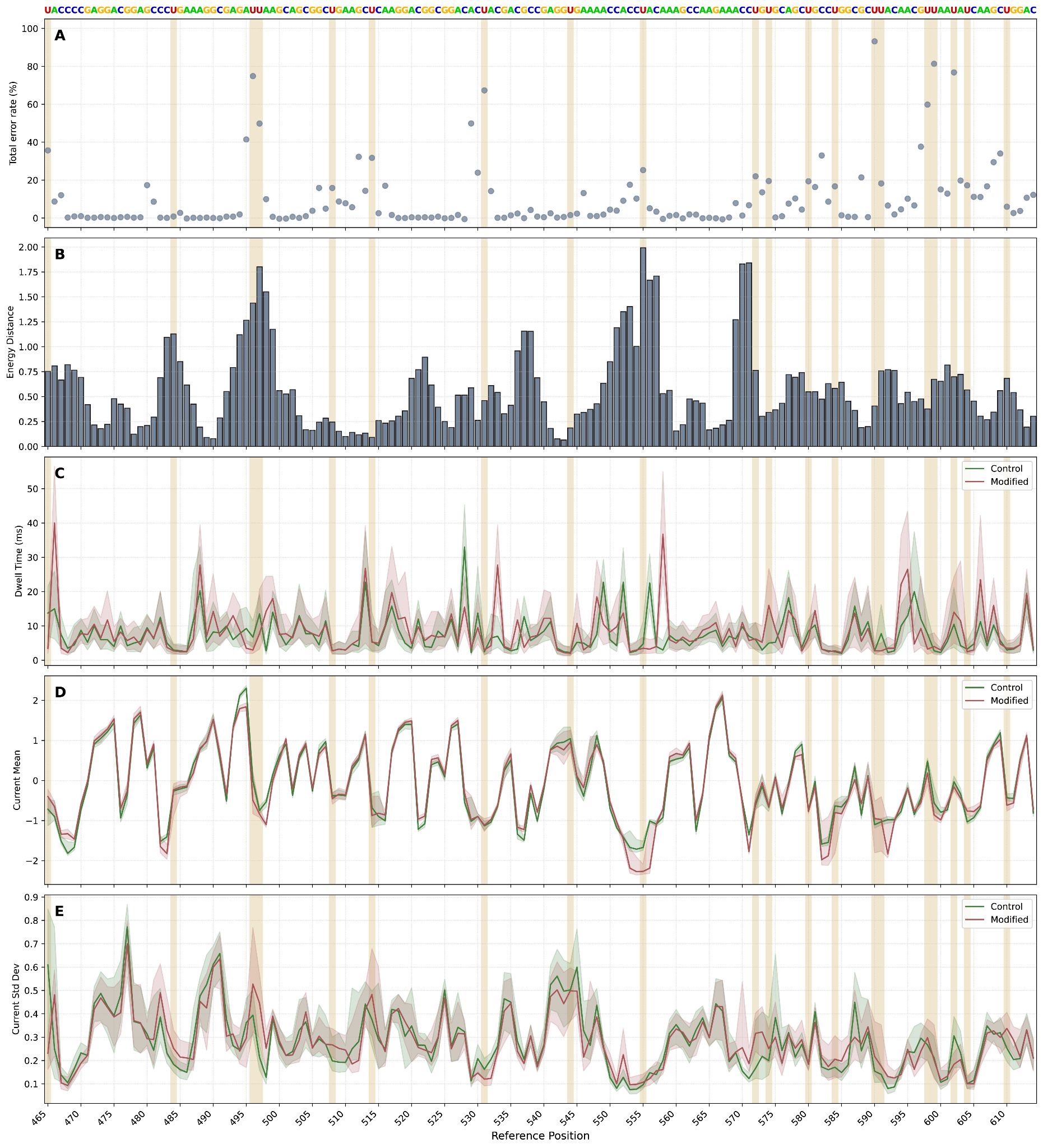


Figure S7. Error and current signatures of RNA modifications in 5moU mCherry mRNA from position 465-615. (A) Total error rate difference between 5moU mCherry mRNA and canonical mRNA. (B) Energy distance per position between 5moU mCherry mRNA and canonical mRNA. Breakdown of current-level metrics: (C) Dwell time (D) Normalized current mean (E) Current standard deviation. In all dwell time, current mean, and current standard deviation plots, the solid line represents the median across all reads, with the shaded area indicating the 25th to 75th percentile range. The highlighted regions indicate the modification sites, and the letters above the total error rate plots represent the reference sequence. Raw current signal alignment is performed by Uncalled4.


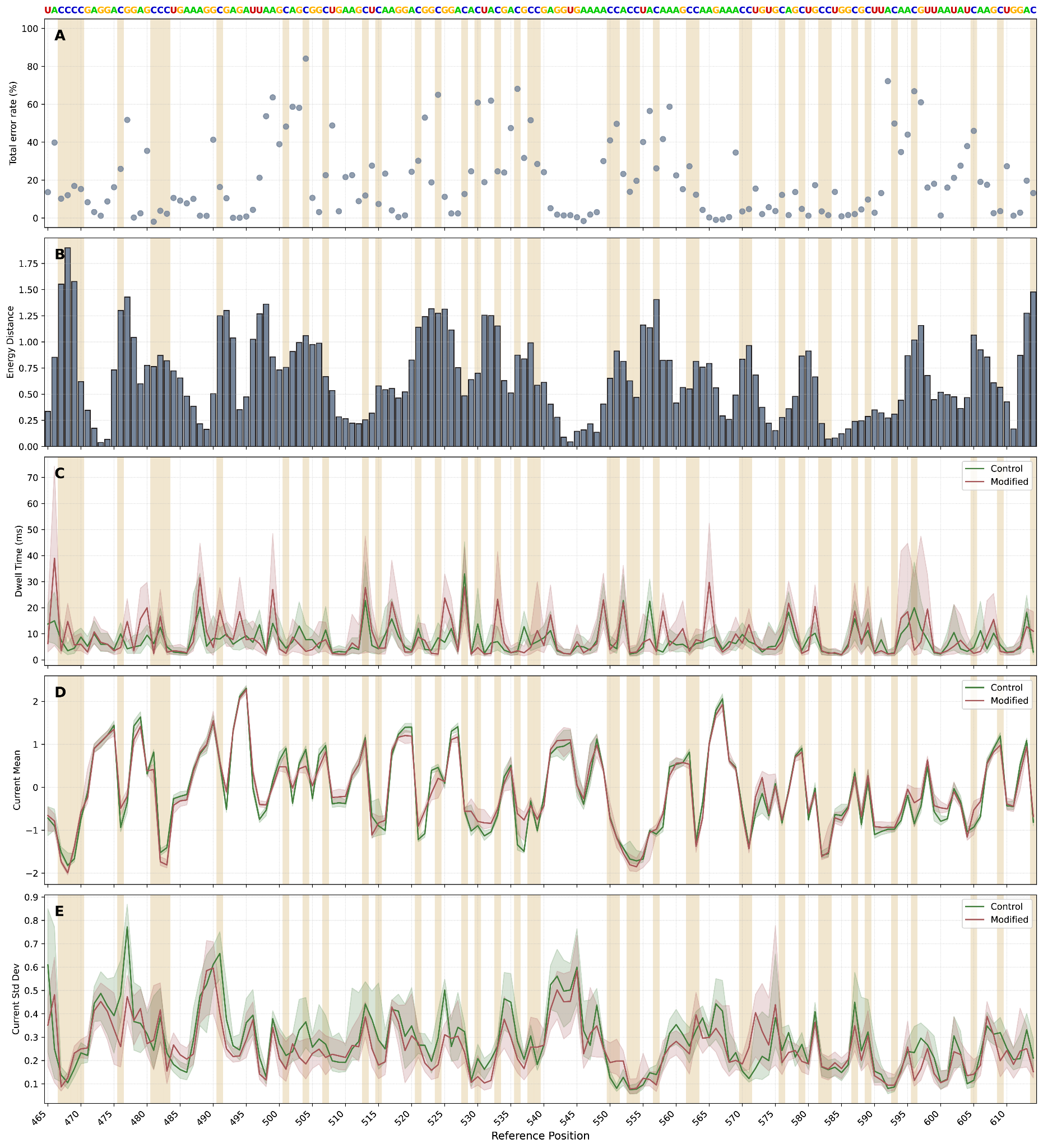


Figure S8. Error and current signatures of RNA modifications in 5iodoC mCherry mRNA from position 465-615. (A) Total error rate difference between 5iodoC mCherry mRNA and canonical mRNA. (B) Energy distance per position between 5iodoC mCherry mRNA and canonical mRNA. Breakdown of current-level metrics: (C) Dwell time (D) Normalized current mean (E) Current standard deviation. In all dwell time, current mean, and current standard deviation plots, the solid line represents the median across all reads, with the shaded area indicating the 25th to 75th percentile range. The highlighted regions indicate the modification sites, and the letters above the total error rate plots represent the reference sequence. Raw current signal alignment is performed by Uncalled4.

## Section 3. Modification aware basecaller


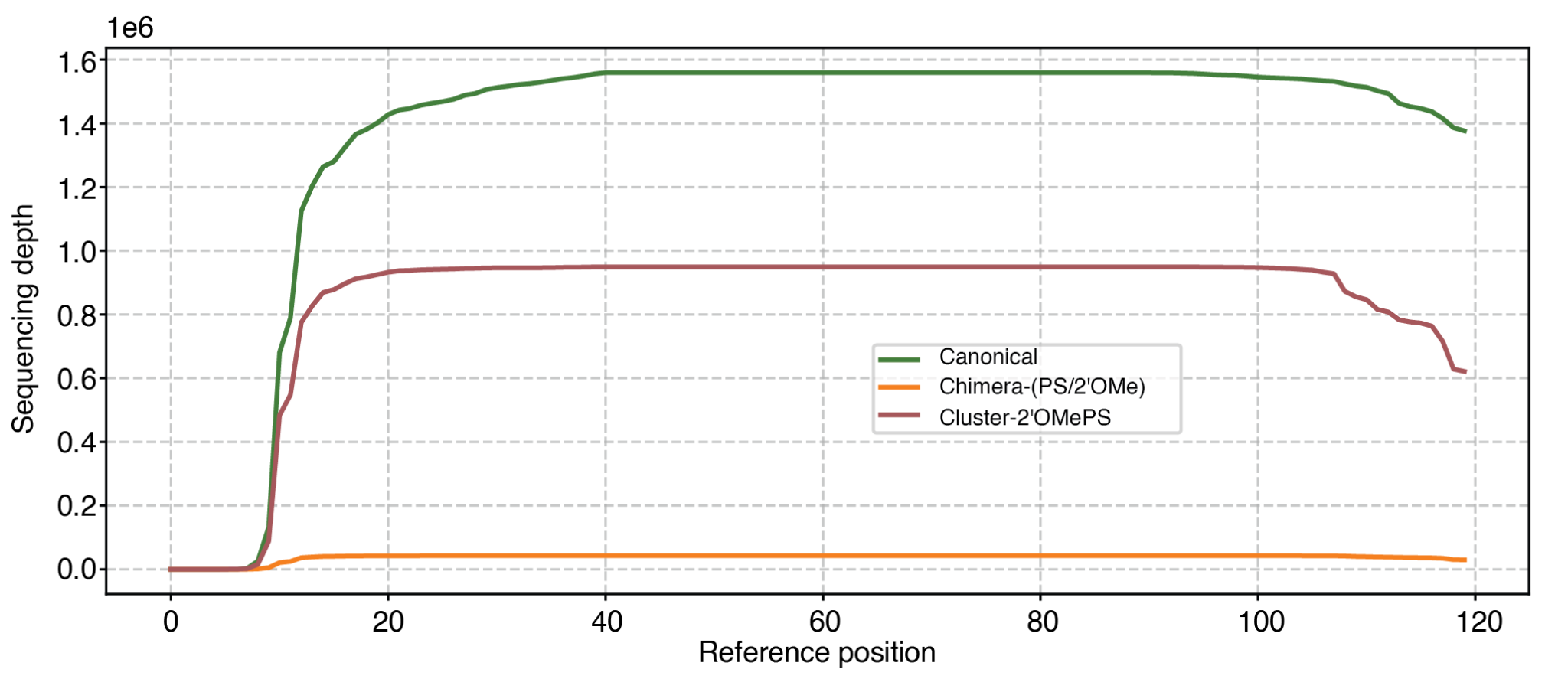


Figure S9. Sequencing depth of Canonical, Chimera-(PS/2′OMe), Cluster-2′OMePS. Data are basecalled using corresponding modification aware basecaller (For example rna004_130bps_sup@v5.2.0_inosine_m6A_2OmeA@v1 for Chimera-(PS/2′OMe)). Reads are filtered to exclude secondary and unmapped sequences, retaining only uniquely mapped reads with a minimum mapping quality of Q20.


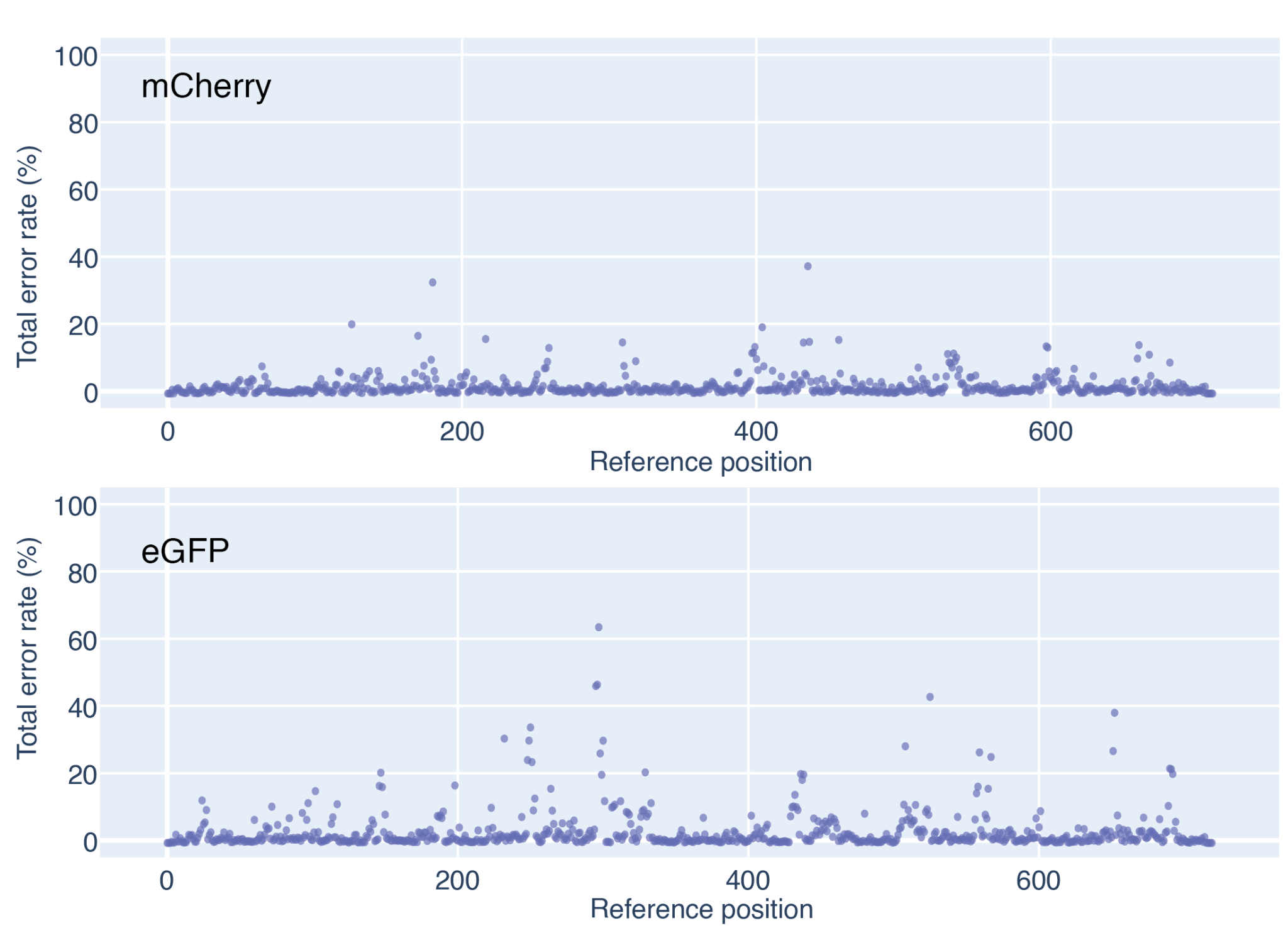


Figure S10. The total error rate of the m5C mCherry and eGFP IVT mRNAs base called by rna004_130bps_sup@v5.1.0_m5C@v1. Most of the mRNAs are correctly called, with the total error rate under 20% in most cases and few positions exceeding 30%.


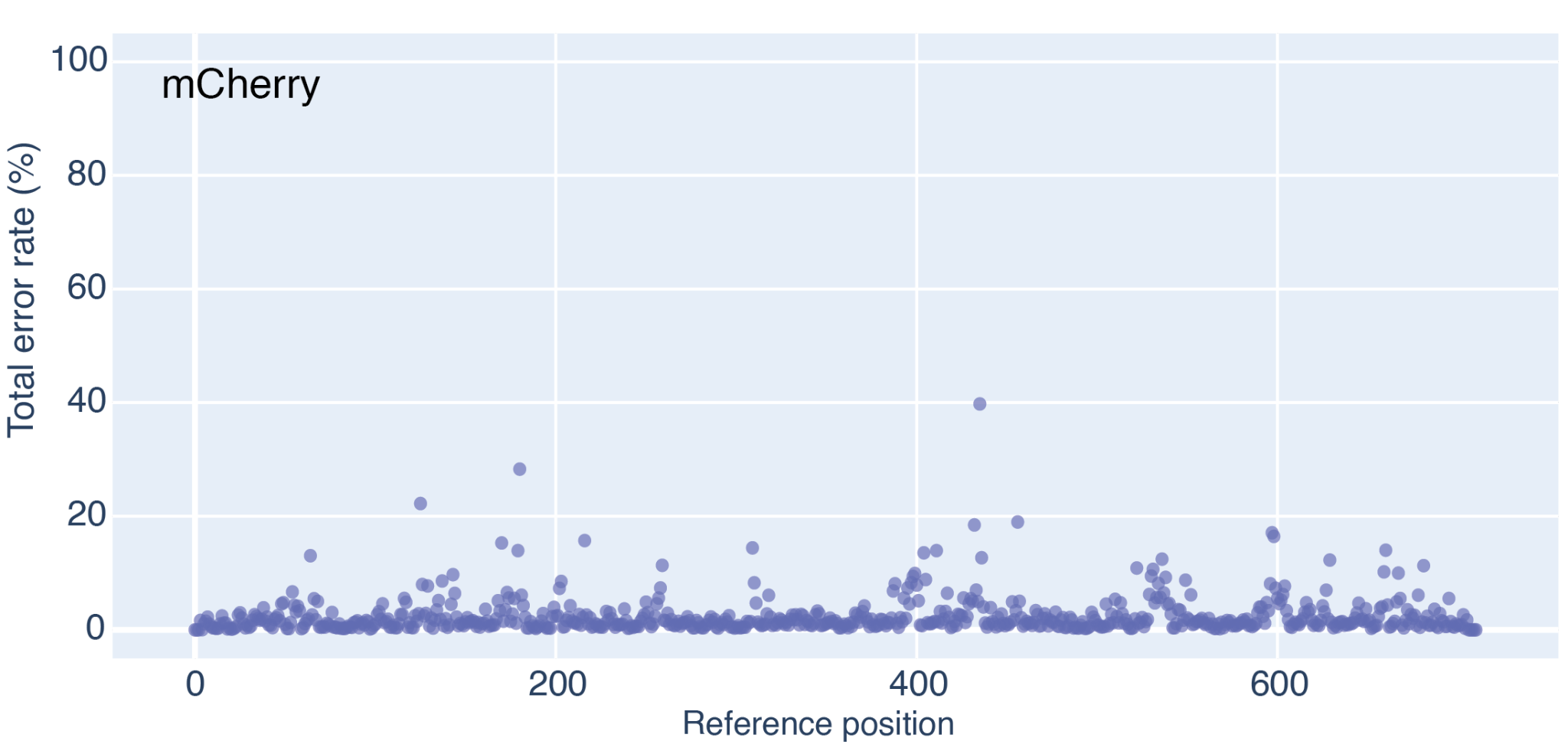


Figure S11. The total error rate of the m5C mCherry IVT mRNAs base called by rna004_130bps_sup@v5.2.0_m5C_2OmeC@v1. Most of the mRNAs are correctly called, with the total error rate under 20% in most cases and few positions exceeding 30%.


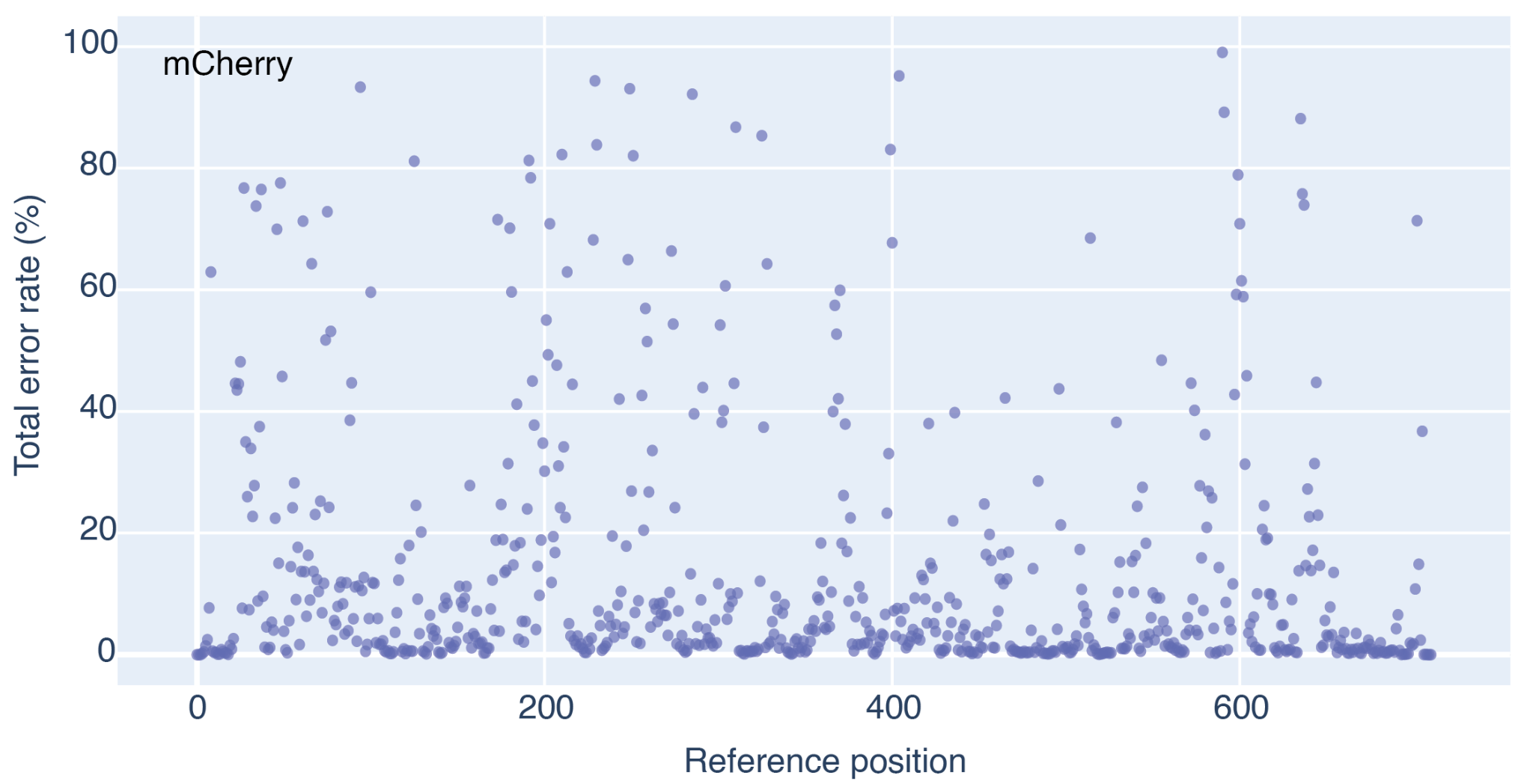


Figure S12. The total error rate of the N1MeΨ mCherry IVT mRNAs base called by rna004_130bps_sup@v5.1.0_pseU@v1. The overall error rate is high.


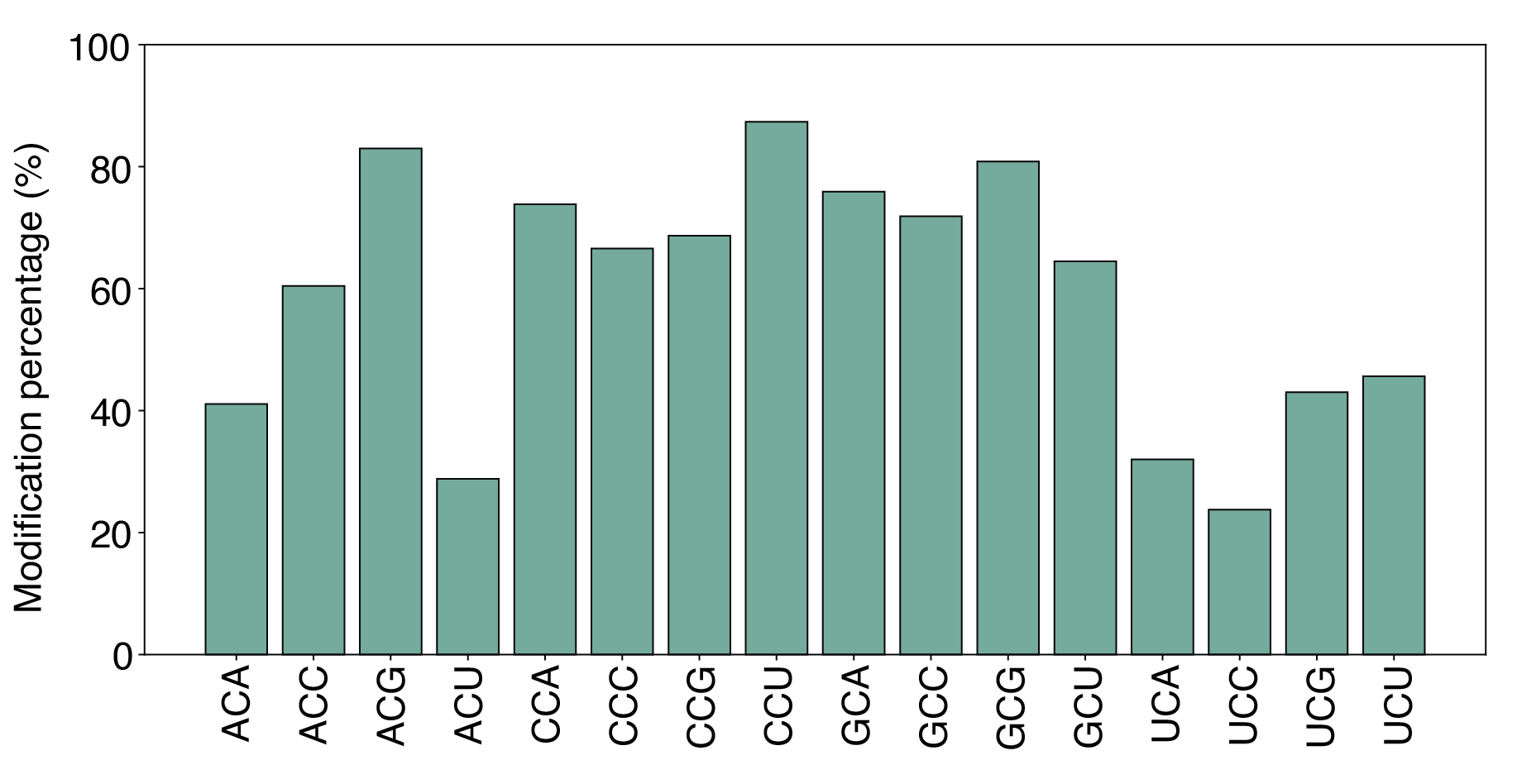


Figure S13. Analysis of the influence of 3 nt kmer on the modification percentage for m5C modified mCherry and eGFP. The presence of a uracil immediately preceding an m5C site appeared to correlate with a lower modification call rate. The basecalling model used here is rna004_130bps_sup@v5.1.0_m5C@v1.


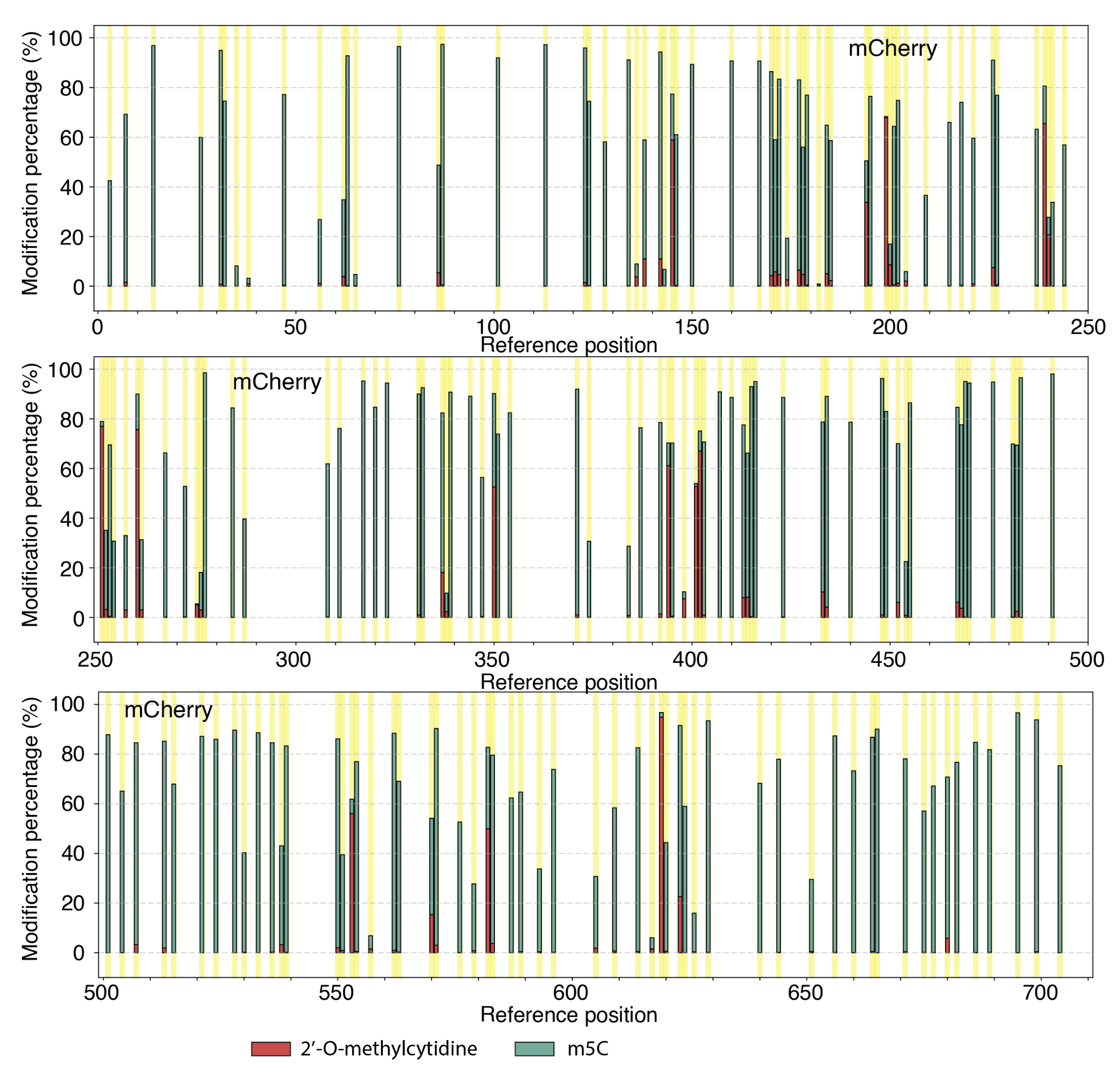


Figure S14. Modification calls for m5C mCherry using the rna004_130bps_sup@v5.2.0_m5C_2OmeC@v1. Highlighted regions are m5C positions.
